# Prevalence of neonatal sepsis and associated factors among neonates admitted in the neonatal intensive care unit at Lira Regional Referral Hospital, Northern Uganda

**DOI:** 10.1371/journal.pone.0315794

**Published:** 2025-01-13

**Authors:** Brendah Katugume, JohnBaptist Muzungu, Nelson Okello, Eustes Kigongo, Deborah Andrinar Namutebi

**Affiliations:** 1 Department of Midwifery, Faculty of Nursing and Midwifery, Lira University, Lira, Uganda; 2 Department of Pediatrics and Child Health, Faculty of Medicine, Lira University, Lira, Uganda; 3 Department of Environmental Health and Disease Control, Faculty of Public Health, Lira University, Lira, Uganda; Bangabandhu Sheikh Mujib Medical University (BSMMU), BANGLADESH

## Abstract

**Background:**

Sepsis is one of the leading causes of mortality and morbidity among neonates. An estimated 5.29–8.73 million Disability-adjusted life years (DALYs) are lost annually in SSA due to neonatal sepsis (NS). Uganda registered stagnated neonatal mortality of 27 deaths per 1000 live births in 2020 of which 12% was attributed to NS. Early risk factor identification and improved obstetric care are proven to reduce deaths due to NS, yet there is scanty literature on the LRRH. We, therefore, determined the prevalence of NS and identified its associated factors within the LRRH of northern Uganda.

**Methods:**

A hospital-based, cross-sectional study with a retrospective chart review was conducted in the neonatal intensive care unit (NICU) at Lira Regional Referral Hospital (LRRH), in northern Uganda. 194 records of neonates admitted to the NICU from September 2022 to February 2023 were reviewed. The participant records were selected by systematic sampling technique and a structured data extraction tool was used to collect data. Using SPSS version 25 data entry and analysis were done. The univariable analysis gave a general description of the data. Logistic regression analysis was used to show associations and the statistical significance was declared at a P value of 0.05 after multivariable analysis.

**Results:**

Among a total of 194 neonates whose charts were reviewed, 80 neonates had neonatal sepsis, giving a proportion of 0.412 and then prevalence of 41.2%. Age in days of the neonate (AOR = 4.212, 95% CI: [1.627-10.903]) for neonates of 1-3days of age, sex where males (AOR = 2.09, 95% CI: [1.123-3.887]), an APGAR score of 1-4 at birth (AOR = 0.309, 95% CI: [0.115-0.831]) and weight at birth <2500g (AOR = 2.543, 95% CI: [1.381-4.683]) were significantly related to it.

**Conclusions and recommendations:**

The results found the prevalence of NS among neonates admitted to the NICU of LRRH high at 41.2%. Factors 1-3 days of age, male sex, a birth weight of <2500g, and an Apgar score of 1-4 at birth among all neonates were significant. Therefore, it was suggested that caregivers ensure safe newborn care, detect infections early, and use prophylactic antibiotics for high-risk babies after birth, to reduce NS. Further research will be conducted on the major causative agents and outcomes of NS in the NICU of LRRH.

## Introduction

A neonate refers to a baby from birth to 28 days of life [1]. Sepsis “is a life-threatening condition that arises when the body’s response to infection causes injury to its own tissues and organs” [2]. Neonatal sepsis (NS) “refers to the systemic response to infection in newborns within the first four weeks following delivery” [3]. “Sepsis is one of the leading causes of mortality and morbidity among neonates…” [4]. Of 5.4 million under-5 mortality cases that occur every year globally, NS accounts for 44% [5]. An estimated 5.29–8.73 million DALYs are lost annually in SSA due to neonatal sepsis (NS) [6]. Uganda registered stagnated neonatal mortality of 27 deaths per 1000 live births [7] with 12% attributed to neonatal sepsis (NS) as of 2019/2020 [7]. “NS is a major cause of morbidity and mortality globally, particularly more common in developing countries as compared to developed countries” [8] Neonatal sepsis and subsequent long-term morbidities such as respiratory failure, pulmonary hypertension, cardiac failure, shock, renal failure, liver function, and cerebral edema were blamed for the loss of 5.3–8.7 million disability-adjusted life years in sub-Saharan Africa alone in 2014 [9, 10].

Neonatal sepsis is categorized into two, that is, Early Onset sepsis (EOS) which is a “disease among neonates aged 72 hours and below” and Late-onset sepsis (LOS) which “occurs from 4 to 28 days” [11]. Neonatal sepsis may develop in utero, through the mother’s genitalia normally EOS, or from the surroundings in a community or hospital which is usually LOS [4, 11]. It has clinical features such as fever, hypothermia, tachycardia, failure to thrive, lethargy, irritability, and listlessness [10]. Furthermore, neonates with sepsis may fail to feed, have convulsions, have fast breathing, severe lower chest indrawing, fever, and hypothermia as the presenting signs [11]. Neonatal sepsis can exhibit mild symptoms at first, but it can quickly advance to meningitis and multisystem organ failure, which are linked to significant mortality and morbidity [12].

With the high occurrence of infectious illnesses and limited access to medical facilities that are sufficiently manned and staffed, neonatal mortality is on the rise in low- and middle-income countries (LMICs) [13]. Numerous variables influenced neonatal sepsis of which birth weight, size at birth, mode of delivery, maturity, Apgar score, and gestational age were found to be the neonatal individual factors [9, 14]; the length of rupture of membranes, maternal age, parity, maternal infection, complications of pregnancy, number of pregnancies and number of antenatal attendances were maternal factors [13]. To combat the rising neonatal mortality due to NS, strategies such as early risk factor identification and improved obstetric care have been implemented. This is done by keeping the area around births clean, and using intrapartum antibiotic prophylaxis, which has been shown to lower the EOS [12]. However, there is a rise in LOS linked to longer hospital stays for preterm infants and higher survival rates [12]. A combined approach to mother and baby care during pregnancy, including instruction on NS causes, warning signs, and preventative measures improves newborn outcomes [12]. Furthermore, delivery and effective care after birth improve newborn outcomes, staff training and education about infection prevention is a crucial step to prevent nosocomial infections [15]. Despite the efforts, NS remains a significant problem in Northern Uganda where 22.2 neonates per 1000 live births suffered sepsis in 2020 [7], which is higher as compared to other parts of the country like Eastern Uganda with 8.9-15 neonates per 1000 live births [16]. The paucity of information about the prevalence of NS in LRRH justified the need for a study to identify the prevalence and factors associated with neonatal sepsis in the hospital.

## Methods and materials

### Study area and period

The study was conducted from March 2023 to July 2023 at Lira Regional Referral Hospital in Northern Uganda. Lira Regional Referral Hospital, which is located in Lira City, serves a population of about 2.2 million people from its mandated catchment districts of the central north which are Amolatar, Apac, Dokolo, Lira, Lira City, Oyam, Kwania, Otuke, Kole, and Alebtong and has a total of 254 beds. LRRH is one of the 13 regional referrals in Uganda, it serves both outpatient and inpatient which includes general and specialized services such as surgery, medicine, dentistry, orthopedics, pediatrics, gynecology and obstetrics care, as well as outpatient services such as immunization, HIV counseling and testing, elimination of mother to child transmission (EMTCT), antenatal care services. The study was carried out in the neonatal intensive care unit which is a subunit under the pediatrics department. NICU has a bed capacity of 36 beds and in 2021 it admitted 1104 neonates and 1224 neonates in 2022. It handles babies with abnormal conditions from tithe me of birth 28 days after birth, among which is neonatal sepsis. This study was conducted in LRRH because it is the main point of contact for neonates born in the Lango subregion. The findings from this study filled the literature gap. They brought awareness of the prevalence and factors associated with neonatal sepsis, and these will direct policymakers about which particular measures to take to improve neonatal health in LRRH.

### Study design and population

This was a hospital-based retrospective chart review cross-sectional study design. The study used records for neonates admitted to the Neonatal Intensive Care Unit (NICU) of Lira Regional Referral Hospital (LRRH) from September 2022 to February 2023.

### Sample size estimation

The sample population was determined using Yamane’s formula of 1973 used for calculating sample size for known populations. Taking the known population size of neonates admitted in the NICU of LRRH from September 2022 to February 2023 from the records of the hospital as follows:

n = N/1+Ne^2^

Where n is the sample size needed, N is the population size (498 records) the counting which was done at the records office on 18^th^ March 2023, as shown in Table 1 below, and e is the margin of error (5%) at 95% confidence level.

**Table 1:** Population of neonates admitted in NICU, LRRH from September 2022 to February 2023.

Month	Year	Admission
September	2022	81
October	2022	88
November	2022	96
December	2022	88
January	2023	60
February	2023	85
**TOTAL**		**498**

n = 498 /1+498(0.05^2^)

n = 222 sample records.

Therefore, the sample size considered was 222 patient records.

### Inclusion criteria

This study included records of all neonates who had been admitted to the neonatal intensive care unit at Lira Regional Referral Hospital from September 2022 to February 2023.

### Exclusion criteria

This study excluded records of all neonates who had been admitted to the neonatal intensive care unit at Lira Regional Referral Hospital from September 2022 to February 2023 but had missing data.

This study excluded repeated records of all neonates who had been admitted in neonatal intensive care unit at lira regional referral hospital from September 2022 and February 2023.

### Ethics approval and consent to participate

Approval to conduct the study was granted by Lira University Research Ethics Committee (LUREC) (LUREC-2023-13), plus a waiver of consent since the retrospective study intended to use participants’ health information with no need to obtain consent from individual patients. Administrative clearance to collect data was obtained from the hospital director of LRRH and later verbal consent was granted by the in-charge NICU at LRRH, and patient files of the neonates were availed to the researcher from 5^th^ May 2023, to 12^th^ May 2023.

### Sampling technique and procedure

The study employed systematic random sampling. The list of neonates that were admitted from September 2022 to February 2023 to NICU. Though the records for all admissions were 498, only 396 were presented with full records. A skip interval (X^th^) was calculated from the total population (N) and the required sample (n) as shown below

X^th^ = N/n

X^th^ = 396/222

X^th^ = 1.78378….

X^th^ ~2

Two papers were folded and a lottery method was used to select one for starting the recruitment, the paper picked had number 2 so all papers that were sorted under number 2 were considered. Thereafter, an interval of 2 was used on the generated list of records until 198 records were obtained. However, of the 198 records 4 had missing information and were left out as per the exclusion criteria. Therefore, a total of 194 records were obtained as shown in Table 2 below.

**Table 2:** Response rate in the study.

Variable	Frequency n (%)
Available	198(89.2)
Cleaned	4(1.8)
Considered	194(87.4)
Missed	24(10.8)
Proposed total	222(100)

### Data collection tool and quality control

Data collection was done using a structured data extraction tool adopted from the Uganda Pediatric Association (UPA) publication indicating the diagnoses that fall under the umbrella of NS [17]. The diagnosis was obtained using the UPA publication because in Uganda it’s the association whose mission is to ensure that children grow up and reach their full potential. The associated factors that were to be studied were adapted from different studies [10, 13, 15, 18–25]. The structured data collection tool had three sections. Section one contained the diagnosis, which during data collection was obtained from the clinical judgment of the doctor who attended to the neonate at the time he/she was admitted. The different diagnoses that fall under the umbrella of neonatal sepsis were listed according to the UPA and if the diagnosis in the patient file matched any of the UPA diagnoses, was a positive diagnosis of neonatal sepsis. Section 2 contained the maternal factor extraction tool which included the parameters: neonate’s ID to identify whose mother is being assessed, maternal age, parity, and number of antenatal attendances. Section 3 contained the neonatal individual factors extraction tool including neonate’s ID, neonate’s age in days, neonate’s gestational age at birth, neonate’s sex, whether there was resuscitation at birth, the mode by which the neonate was delivered, and birth weight.

To ensure quality control, the data collection tool that was used was adapted from previous studies about the prevalence and associated factors of neonatal sepsis which were published in international peer-reviewed journals. The designed data extraction tool was submitted to the supervisor and the LUREC as well as a pediatrician for review and correction to ensure it covers all aspects of the research objectives. The tool was reviewed 2 weeks after designing to confirm it contained the required questions and then was run through the Cronbach alpha to determine its reliability. Internal consistency was ensured by using the same data extraction tool for all patient charts.

The structured data collection tool had clearly defined parameters of the phenomenon under scrutiny and simple words that are easy to understand were used throughout. Data was collected from patient charts of all neonates admitted to the NICU at LRRH from September 2022 to February 2023 that were picked up using the systematic sampling technique and met the inclusion criteria. Data was collected for 8 days by the principal investigator who sat at the NICU table and filled the data extraction tools considering information in patient files. 30 tools per day each taking about 30 minutes were filled and no personal identifier information like names and addresses were collected.

### Data processing and analysis

Data editing and cleaning were performed during data collection, every day after collection, and after entry. Data entry and post-entry cleaning were performed using Microsoft Excel (2013). Data analysis was performed using the Statistical Product and Service Solutions (SPSS) version 25.0 software. Data analysis was done at univariable, bivariable and multivariable levels. Univariable analysis was done to summarize all data as frequencies and proportions for categorical variables, and mean with standard deviation or median with interquartile range for discrete and continuous variables. The prevalence of NS the prevalence of NS was measured using the formula,

$$prevalence=\frac{number\;of\;neonates\;affected\;by\;NS\;from\;September\;2022\;to\;February\;2023}{total\;number\;of\;admitted\;neonates\;in\;NICU\;from\;September\;2022\;to\;February\;2023.}$$

Associations between NS (dependent variable) and each independent variable (maternal and neonatal characteristics) were determined at bivariable analysis. All variables with p<0.2 at bivariable analysis with all other plausible variables from the literature were considered for multivariable analysis. The backward elimination method was performed during multivariable analysis, where all variables were entered into the equation and those that were not statistically significant (p>0.05) dropped one at a time. Adjusted odds ratios and p-values were presented using tables and texts.

## Results

**Fig 1:**
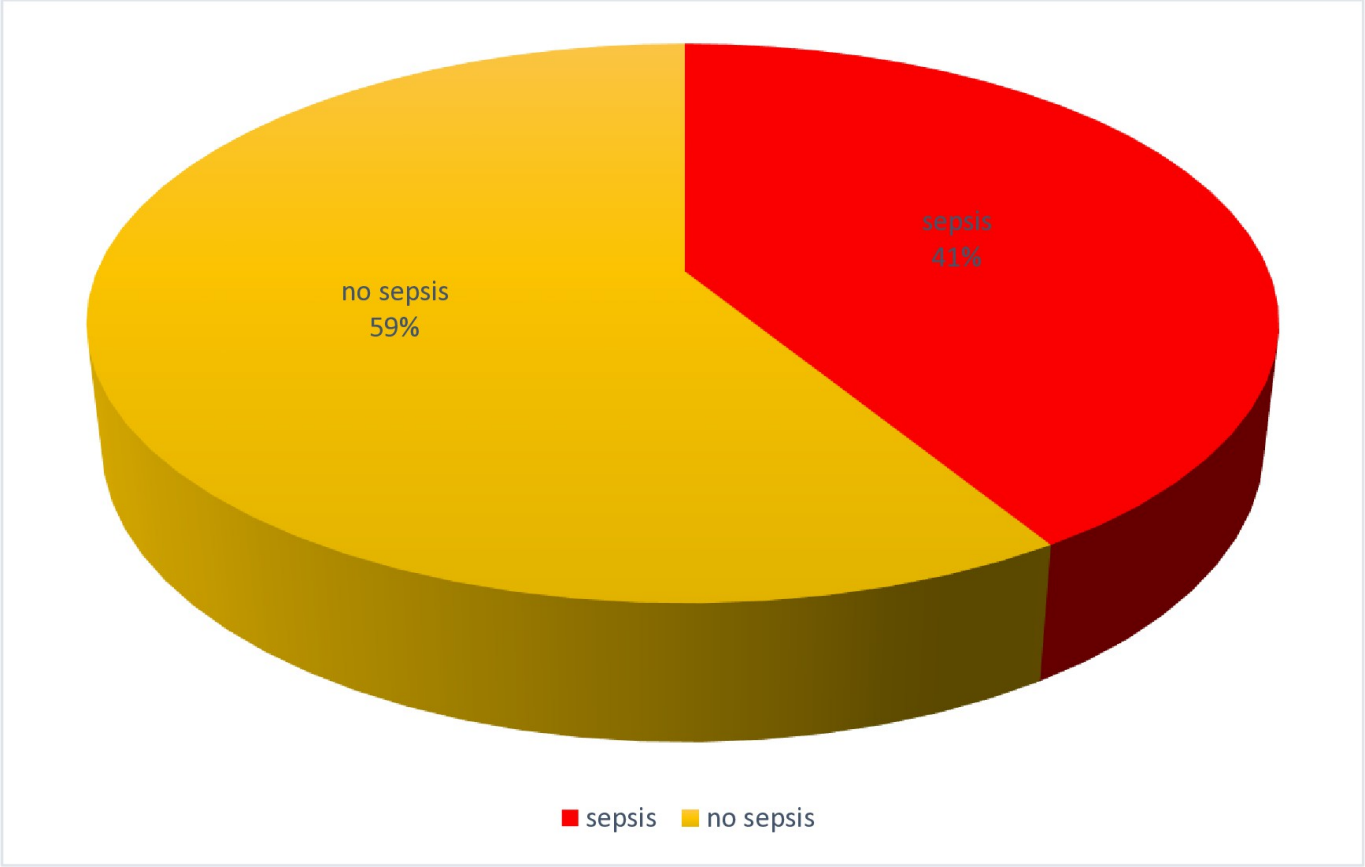
Prevalence of neonatal sepsis.

In this study, the proposed sample size was 222 records of neonates admitted to NICU LRRH from September 2022 to February 2023. Of 389 files present, 194 files were sampled to be considered in the review. This gave a response rate of 87.4% as shown in the table below (Table 2). Among 194 reviewed neonatal charts of neonates admitted to the NICU of LRRH from September 2022 to February 2023, a proportion of 0.412(80) neonates had suffered sepsis (41.2%) of the reviewed charts, and 0.58(114) neonates (58.8%) had not suffered sepsis (Fig 1).

### Maternal characteristics

Table 3 shows that the majority of the mothers (n = 87, 44.8%) to the neonates whose charts were involved in the study were para 2-4, and the majority and almost a half (n = 91, 46.9%) were 20-25 years of age, and more than a half (n = 113, 58.2%), had attended ANC >4 times. All of the maternal factors are summarized in Table 3 below.

**Table 3:** Frequencies of the maternal factors associated with neonatal sepsis among neonates admitted at NICU of LRRH at univariate analysis.

Variable	Frequency n (%)
Mother’s parity	
1	86(44.3)
2-4	87(44.8)
>/=5	21(10.8)
Maternal age	
<20	44(22.7)
20-25	91(46.9)
26-35	49(25.3)
>35	10(5.2)
Number of ANC attendances	
1-4	81(41.8)
>4	113(58.2)

### Neonatal characteristics

Table 4 indicates that more than a third of the neonates (n = 169, 87.1%) were in a range of 1-3 days of age, half (50.5%) were born before 37 completed weeks of gestation, a half (n = 99, 51%) were males, more than a half (n = 112, 57.7%) had been resuscitated at birth, almost three quarters (n = 130, 67%) were born vaginally, slightly more than a half (n = 105, 54.1%) had a birth weight of <2500g, and 53.1% (n = 103) had an Apgar score of 8-10.

**Table 4:** Frequencies of neonatal individual factors associated with neonatal sepsis among neonates admitted at NICU of LRRH at univariate analysis.

Variable	Frequency n (%)
Age in days	
1-3	169 (87.1)
4-28	25 (12.9)
Gestational age at birth	
<37	98 (50.5)
>/-=37	96 (4.5)
sex	
Male	99 (51.0)
Female	95 (49.0)
Resuscitation at birth	
Yes	112 (57.7)
No	82 (42.3)
Mode of delivery	
Vaginal	130 (67.0)
Assisted vaginal	6 (3.1)
Cesarean section	58 (29,9)
Birth weight in grams	
<2500	105 (54.1)
>/=2500	89 (45.9)
APGAR score	
1-4	24 (12.4)
5-7	67 (34.5)
8-10	103 (53.1)

### Factors associated with neonatal sepsis

Table 5 shows that age in days was significantly associated with neonatal sepsis (COR 3.575 [1.459-8.762]). Gestational age at birth was positively related to neonatal sepsis (COR 2.062 [1.153-3.690]). The logistic regression showed that NS was also significantly related to birthweight (COR 2.442 [1.359-4.385]). Apgar score too showed significance about NS (COR O.257 [0.100-0.660]). The results of binary logistic regression between individual factors and NS are shown in Table 5 below.

**Table 5:** Results of factors associated with neonatal sepsis among neonates admitted in NICU of LRRH, Lira City, Northern Uganda from September 2022 to February 2023.

Variable	Outcome n (%)		P-value	COR (95% CI)	p-value	AOR (95% CI)
	Sepsis	No sepsis				
Maternal parity						
1	35(40.7)	51(59.1)	0.309	0.583(0.206-1.649)		
2-4	39(44.8)	48(55.2)	0.18	0.492(0.115-1.388)		
>=5	6(28.6)	15(71.4)				
Maternal age						
<20 years	17(38.6)	27(61.4)	0.611	0.681(0.155-2.997)		
20-25 years	42(46.2)	49(53.8)	0.337	0.500(0.122-2.056)		
26-35 years	18(36.7)	31(63.3)	0.686	0.738(0.169-3.216)		
>35years	3(30.0)	7(70.0)				
ANC attendance						
1-4 times	32(39.5)	49(60.5)	0.678	1.131(0.632-2.022)		
>4 times	48(42.5)	65(57.5)				
Age in days						
1-3 days	63(37.3)	106(62.7)	0.005	3.575(1.459-8.762)	0.004	4.159(1.583-10.925)
4-28 days	17(68)	8(32)				
Gestational age at birth						
<37 weeks	32(32.7)	66(67.3)	0.015	2.062(1.153-3.690)		
>=37 weeks	48(50)	48(50)				
Sex						
Male	34(35.4)	64(64.6)	0.062	1.732(0.972-3.085)	0.047	1.894(1.009-3.553)
Female	46(47.4)	50(52.6)				
Resuscitation at birth						
Yes	50(44.6)	62(55.4)	0.261	0.715(0.399-1.282)		
No	30(36.6)	52(63.4)				
Mode of delivery						
Vaginal	54(41.5)	76(58.5)	0.808	0.925(0.492-1.739)		
Assisted vaginal	3(50)	3(50)	0.625	0.657(0.122-3.542)		
Cesarean section	23(39.7)	35(60.3)				
Birth weight at birth						
<2500 grams	33(31.4)	72(68.6)	0.003	2.442(1.359-4.385)	0.199	1.758(0.744-4.156)
>=2500 grams	47(52.8)	42(47.2)				
Apgar score						
1-4	16(66.7)	8(33.3)	0.005	0.257(0.100-0.660)	0.02	0.309(0.115-0.831)
5-7	29(43.3)	38(50.7)	0.222	0.674(0.358-1.269)	0.196	0.674(0.358-1.269)
8-10	35(34)	68(66)				

The variables with a P-value of less than 0.2 at bivariate analysis were considered for multivariable analysis. With reference from bivariable analysis, age in days, Gestational age at birth, birthweight, sex and Apgar score had a P value of less than 0.2 and were considered for multivariable analysis. Therefore, the analysis proved that neonate’s age in days, neonate sex, birth weight and APGAR score had significant relationship with neonatal sepsis as shown in Table 5 below.

## Discussion

The prevalence of neonatal sepsis among neonates admitted to the NICU of LRRH from September 2022 to January 2023 is 41.2% a proportion of 0.41 (80 neonates of the reviewed 194 records). indicating that close to half of the neonates admitted to LRRH NICU suffer NS. This could be due to inadequate knowledge among health workers, poor handling of these neonates, use of unsterilized equipment to care for the neonates, or even colonization of the NS causing microbes in the NICU. This then requires a refreshment of knowledge of handling neonates and cabbing the raised prevalence of NS.

The prevalence of neonatal sepsis among neonates admitted to the NICU of LRRH from September 2022 to January 2023 is 41.2% a proportion of 0.41 (80 neonates of the reviewed 194 records). The results are similar to those of studies conducted in north and east Ethiopia (45%) as well as the global prevalence of 48% [15]. However, this prevalence is higher than that of Northern Uganda (17.7%) [7], Kenya (23.9%), Nigeria (18.2%), Tanzania (31.4%), India (7.6%), and Iran(15.98%) [15, 24, 26]; and lower than that of southern Ethiopia (78.3%) [15]. The difference could be due to differences in socio-demographics in study areas and the way the results regarding diagnosis were obtained. While some of the studies were conducted in regions, others covered the whole country and others were global, this study was conducted specifically at Lira Regional Referral Hospital of Northern Uganda. Different studies considered different methodologies to obtain the diagnosis of NS while this study considered the umbrella diagnoses and clinical judgment for the diagnosis of NS as per APA to obtain the diagnosis [17] because the other methods were not used as diagnostic tools within LRRH. The difference in prevalence in the same area may be due to the difference in time of conducting the study as the former was in 2021 [7] and it also considered the whole of northern Uganda while the current study considered only Lira regional referral hospital.

The results that a low birth weight of <2500g was significantly related to NS build on the existing evidence of the positive relationship between neonatal sepsis and low birth weight in that neonates who are born with low birth weight are more likely to develop NS as compared to those born with normal birth weight. This finding is similar to those of a study conducted in Ethiopia as a whole and eastern Ethiopia singly [15, 23]. This may be because low birth weight babies have low subcutaneous fat which predisposes them to hypothermia due to high surface area to volume ratio (weight ratio) and have a compromised immune system which predisposes them to NS because of low immunity.

In this study, the age of neonates in days was significantly associated with neonatal sepsis which is similar to a systematic review and meta-analysis study conducted in Ethiopia which reported that neonatal sepsis is more common among neonates in less than 7 days of life [27]. This may be because neonates are at a higher risk of infection since they are developmentally weak and have immature immune systems.

These findings show that there is a positive association between a lower than 7 APGAR score and neonatal sepsis and this is similar to the findings of studies conducted in Eastern Ethiopia and in all public hospitals in Ethiopia [15, 23]. This may be because the neonates with low APGAR scores are believed to have been exposed to infection-causing microbes at birth. This is through unsterile resuscitation equipment such as bulb syringes which may introduce microorganisms to the lungs and an unsafe environment as they intend to save lives [13]. This then predisposes the neonates born with very low Apgar scores to a risk of infections like neonatal sepsis as compared to the counter neonates’ functioning.

### Strength, limitation and limitation mitigation of the study

#### Strength

Recruiting neonates from Lira Regional Referral Hospital NICU provided a good representation of neonates with neonatal sepsis in the Lango subregion since it is the only intensive care unit and it is main center of referral of sick neonates in the entire subregion.

#### Limitation

The recorded number of admissions in records (498) did not match the files that were present (396) and therefore the proposed sample size was not met, only 198 patient files were accessed of which 4 had incomplete information therefore, were left out, and finally 194 patient files considered in the study.

### Limitation mitigation

The actual sample size which was obtained was adequate to produce statistical significance of the results of the study.

The files with missing information that were left out were few hence they could not affect the quality of the study results.

## Conclusion and recommendations

There was still an unreported prevalence of NS in Lira Regional Referral Hospital. The prevalence of NS among neonates admitted to the NICU of LRRH which is the major health facility and point of contact for most mothers and neonates in the Lango subregion was high at 41.2%. Neonate being 1-3 days of age, being of a male sex, having a low birth weight (weight <2500g), and having an Apgar score of 1-4 at birth was significantly associated with NS. Therefore, in practice health care providers should adhere to precautions while performing procedures on all neonates. Counseling of mothers during ANC visits about safe newborn care by healthcare providers need to be emphasized. Furthermore, early detection and prevention of infection should be considered among neonates after birth by healthcare providers. For all neonates already considered to be exposed, prophylactic antibiotics after stabilization would reduce the risk of developing NS. Recommendations for further studies are about the major causative agents of NS in LRRH to help identify which particular antibiotics would best improve neonatal outcomes of those at risk of NS.

## Supporting information

S1 Dataset (DOCX)

## Abbreviations

NS: Neonatal Sepsis
DALYs: Disability-adjusted life years
SSA: Sub-Sahara Africa
WHO: World Health Organization
EOS: Early Onset Sepsis
LOS: Late Onset Sepsis
EONS: Early Onset Neonatal Sepsis
LONS: Late Onset Neonatal Sepsis
LRRH: Lira Regional Referral Hospital
NICU: Neonatal Intensive Care Unit
UN: United Nations
PROM: Premature Rupture of Membranes
ANC: Antenatal Care
UNICEF: United Nations International Children’s Emergency Fund
Ubos: Uganda Bureau of Standards
SDG 3: Sustainable Development Goal 3
UHDS: Uganda Demographics Survey
OR: Odds Ratio
CI: Confidence Interval
COR: Crude Odds Ratio
AOR: Adjusted Odds Ratio
