# Supplementary material for: Prevalence of neonatal sepsis and associated factors among neonates admitted in the neonatal intensive care unit at Lira Regional Referral Hospital, Northern Uganda
Source: PLoS One. 2025 Jan 13;20(1):e0315794. doi: 10.1371/journal.pone.0315794 (PMC11730428; doi:10.1371/journal.pone.0315794)
Supplement: S1 Dataset — (DOCX) [file pone.0315794.s001.docx]

| A1 | Numeric | 8 | 0 | Category/diagnosis | {1, presumed sepsis}... | None | 8 | Right | Nominal |
| --- | --- | --- | --- | --- | --- | --- | --- | --- | --- |
| B1 | Numeric | 8 | 0 | Mother's parity | {1, 1}... | None | 8 | Right | Nominal |
| B2 | Numeric | 8 | 1 | Maternal age | {1.0, <20}... | None | 8 | Right | Ordinal |
| B3 | Numeric | 8 | 0 | Number of ANC attendances | {1, 1-4}... | None | 8 | Right | Nominal |
| C1 | Numeric | 8 | 1 | Age in days | {1.0, 1-3}... | None | 8 | Right | Ordinal |
| C2 | Numeric | 8 | 1 | Gestational age at birth | {1.0, <37}... | None | 8 | Right | Ordinal |
| C3 | Numeric | 8 | 0 | Sex | {1, Male}... | None | 8 | Right | Nominal |
| C4 | Numeric | 8 | 0 | Resuscitation at birth | {1, Yes}... | None | 8 | Right | Nominal |
| C5 | Numeric | 8 | 0 | Mode of delivery | {1, Vaginal}... | None | 8 | Right | Nominal |
| C6 | Numeric | 8 | 1 | Birth weight in grams | {1.0, <2500}... | None | 8 | Right | Ordinal |
| C7 | Numeric | 8 | 1 | Apgar score | {1.0, 1-4}... | None | 8 | Right | Ordinal |
| OUTCOME | Numeric | 8 | 0 | SEPSIS | {0, SEPSIS}... | None | 8 | Right | Nominal |

| A1 | B1 | B2 | B3 | C1 | C2 | C3 | C4 | C5 | C6 | C7 | C8 |
| --- | --- | --- | --- | --- | --- | --- | --- | --- | --- | --- | --- |
| Other illnesses | 2-4 | 26-35 | 1-4 | 1-3 | <37 | Male | No | Caesarean section | <2500 | 5-7 | NO SEPSIS |
| Early-onset sepsis | 1 | <20 | >4 | 1-3 | >/-=37 | Male | Yes | Caesarean section | >/=2500 | 5-7 | SEPSIS |
| Early-onset sepsis | 1 | <20 | >4 | 1-3 | >/-=37 | Female | Yes | Caesarean section | >/=2500 | 5-7 | SEPSIS |
| Early-onset sepsis | 1 | <20 | 1-4 | 1-3 | <37 | Female | Yes | Vaginal | <2500 | 1-4 | SEPSIS |
| presumed sepsis | 1 | 20-25 | >4 | 1-3 | >/-=37 | Female | Yes | Vaginal | >/=2500 | 8-10 | SEPSIS |
| Other illnesses | 1 | 20-25 | >4 | 1-3 | >/-=37 | Male | Yes | Vaginal | >/=2500 | 8-10 | NO SEPSIS |
| presumed sepsis | 1 | <20 | 1-4 | 1-3 | <37 | Male | No | Vaginal | <2500 | 5-7 | SEPSIS |
| presumed sepsis | 1 | <20 | 1-4 | 1-3 | <37 | Female | No | Vaginal | <2500 | 1-4 | SEPSIS |
| Other illnesses | >/=5 | >35 | 1-4 | 1-3 | >/-=37 | Male | Yes | Vaginal | >/=2500 | 8-10 | NO SEPSIS |
| Early-onset sepsis | >/=5 | 26-35 | 1-4 | 1-3 | <37 | Female | Yes | Vaginal | <2500 | 5-7 | SEPSIS |
| Early-onset sepsis | 1 | <20 | >4 | 1-3 | >/-=37 | Male | No | Vaginal | >/=2500 | 8-10 | SEPSIS |
| Other illnesses | 2-4 | 20-25 | >4 | 1-3 | >/-=37 | Female | No | Vaginal | >/=2500 | 5-7 | NO SEPSIS |
| Early-onset sepsis | 1 | 26-35 | 1-4 | 1-3 | <37 | Female | No | Vaginal | <2500 | 8-10 | SEPSIS |
| Other illnesses | 1 | 20-25 | >4 | 1-3 | >/-=37 | Male | Yes | Vaginal | >/=2500 | 8-10 | NO SEPSIS |
| presumed sepsis | 2-4 | 26-35 | >4 | 1-3 | >/-=37 | Male | Yes | Caesarean section | >/=2500 | 8-10 | SEPSIS |
| Early-onset sepsis | >/=5 | 26-35 | 1-4 | 1-3 | <37 | Female | Yes | Caesarean section | <2500 | 5-7 | SEPSIS |
| Other illnesses | 2-4 | 20-25 | 1-4 | 1-3 | <37 | Male | No | Vaginal | <2500 | 8-10 | NO SEPSIS |
| Other illnesses | >/=5 | >35 | 1-4 | 1-3 | <37 | Female | No | Caesarean section | <2500 | 5-7 | NO SEPSIS |
| Other illnesses | >/=5 | 26-35 | 1-4 | 1-3 | >/-=37 | Female | No | Vaginal | <2500 | 5-7 | NO SEPSIS |
| Other illnesses | 1 | 20-25 | >4 | 1-3 | <37 | Male | No | Caesarean section | <2500 | 5-7 | NO SEPSIS |
| Other illnesses | 1 | <20 | 1-4 | 1-3 | >/-=37 | Male | Yes | Vaginal | >/=2500 | 8-10 | NO SEPSIS |
| presumed sepsis | 2-4 | 26-35 | 1-4 | 1-3 | <37 | Male | No | Vaginal | <2500 | 5-7 | SEPSIS |
| Other illnesses | 2-4 | 20-25 | >4 | 1-3 | >/-=37 | Female | No | Vaginal | <2500 | 8-10 | NO SEPSIS |
| Early-onset sepsis | 2-4 | 20-25 | 1-4 | 4-28 | <37 | Male | No | Caesarean section | >/=2500 | 1-4 | SEPSIS |
| Early-onset sepsis | 2-4 | 26-35 | >4 | 4-28 | <37 | Male | No | Vaginal | <2500 | 1-4 | SEPSIS |
| Early-onset sepsis | 1 | 20-25 | >4 | 1-3 | >/-=37 | Male | No | Caesarean section | >/=2500 | 1-4 | SEPSIS |
| presumed sepsis | 2-4 | <20 | >4 | 1-3 | <37 | Female | No | Caesarean section | <2500 | 1-4 | SEPSIS |
| Other illnesses | 1 | 20-25 | >4 | 1-3 | >/-=37 | Male | Yes | Vaginal | >/=2500 | 1-4 | NO SEPSIS |
| Other illnesses | 2-4 | 20-25 | >4 | 1-3 | >/-=37 | Male | Yes | Vaginal | >/=2500 | 5-7 | NO SEPSIS |
| presumed sepsis | 1 | 20-25 | 1-4 | 1-3 | <37 | Male | Yes | Vaginal | >/=2500 | 5-7 | SEPSIS |
| Early-onset sepsis | 2-4 | 26-35 | >4 | 1-3 | >/-=37 | Female | Yes | Vaginal | <2500 | 8-10 | SEPSIS |
| presumed sepsis | 1 | <20 | 1-4 | 1-3 | >/-=37 | Female | Yes | Vaginal | <2500 | 1-4 | SEPSIS |
| Early-onset sepsis | 2-4 | <20 | >4 | 1-3 | >/-=37 | Female | No | Caesarean section | >/=2500 | 1-4 | SEPSIS |
| Meningitis | 1 | 20-25 | >4 | 4-28 | >/-=37 | Male | Yes | Vaginal | >/=2500 | 8-10 | SEPSIS |
| presumed sepsis | >/=5 | 26-35 | >4 | 1-3 | <37 | Female | Yes | Caesarean section | <2500 | 5-7 | SEPSIS |
| Other illnesses | 2-4 | 20-25 | >4 | 1-3 | <37 | Male | Yes | Assisted vaginal | <2500 | 8-10 | NO SEPSIS |
| presumed sepsis | 2-4 | 26-35 | >4 | 1-3 | >/-=37 | Female | Yes | Caesarean section | >/=2500 | 8-10 | SEPSIS |
| Other illnesses | 1 | 20-25 | >4 | 1-3 | >/-=37 | Male | Yes | Vaginal | >/=2500 | 8-10 | NO SEPSIS |
| Other illnesses | 1 | 20-25 | 1-4 | 1-3 | <37 | Female | No | Vaginal | <2500 | 8-10 | NO SEPSIS |
| Early-onset sepsis | 1 | 26-35 | >4 | 4-28 | >/-=37 | Female | No | Caesarean section | >/=2500 | 8-10 | SEPSIS |
| presumed sepsis | 1 | <20 | 1-4 | 1-3 | <37 | Female | Yes | Caesarean section | >/=2500 | 8-10 | SEPSIS |
| Other illnesses | 2-4 | <20 | >4 | 1-3 | >/-=37 | Female | No | Vaginal | >/=2500 | 8-10 | NO SEPSIS |
| Early-onset sepsis | 1 | 20-25 | >4 | 1-3 | >/-=37 | Female | No | Vaginal | <2500 | 5-7 | SEPSIS |
| presumed sepsis | 2-4 | 20-25 | 1-4 | 1-3 | <37 | Male | Yes | Caesarean section | <2500 | 5-7 | SEPSIS |
| Other illnesses | 2-4 | 26-35 | >4 | 1-3 | >/-=37 | Female | Yes | Vaginal | <2500 | 5-7 | NO SEPSIS |
| Other illnesses | 1 | <20 | >4 | 1-3 | <37 | Female | No | Vaginal | <2500 | 5-7 | NO SEPSIS |
| Other illnesses | >/=5 | 26-35 | 1-4 | 1-3 | <37 | Female | Yes | Caesarean section | <2500 | 5-7 | NO SEPSIS |
| Other illnesses | 2-4 | 26-35 | 1-4 | 1-3 | <37 | Female | No | Caesarean section | <2500 | 5-7 | NO SEPSIS |
| Early-onset sepsis | 2-4 | 20-25 | 1-4 | 1-3 | >/-=37 | Female | No | Caesarean section | >/=2500 | 5-7 | SEPSIS |
| Other illnesses | 2-4 | 20-25 | >4 | 1-3 | <37 | Female | Yes | Caesarean section | <2500 | 5-7 | NO SEPSIS |
| Other illnesses | 1 | <20 | 1-4 | 1-3 | <37 | Male | No | Vaginal | <2500 | 8-10 | NO SEPSIS |
| Other illnesses | 2-4 | 20-25 | 1-4 | 1-3 | >/-=37 | Male | Yes | Vaginal | >/=2500 | 8-10 | NO SEPSIS |
| Other illnesses | 2-4 | 20-25 | >4 | 1-3 | <37 | Male | No | Vaginal | <2500 | 8-10 | NO SEPSIS |
| Other illnesses | 1 | 26-35 | 1-4 | 1-3 | <37 | Male | Yes | Assisted vaginal | >/=2500 | 1-4 | NO SEPSIS |
| Other illnesses | 1 | 26-35 | 1-4 | 1-3 | >/-=37 | Female | Yes | Caesarean section | >/=2500 | 8-10 | NO SEPSIS |
| Necrotizing enterocolitis | 2-4 | 26-35 | >4 | 1-3 | >/-=37 | Female | Yes | Vaginal | >/=2500 | 8-10 | SEPSIS |
| Necrotizing enterocolitis | 1 | 20-25 | 1-4 | 1-3 | <37 | Male | No | Vaginal | <2500 | 8-10 | SEPSIS |
| Other illnesses | 1 | 20-25 | >4 | 1-3 | <37 | Male | No | Vaginal | <2500 | 8-10 | NO SEPSIS |
| Other illnesses | 1 | 26-35 | >4 | 1-3 | <37 | Male | Yes | Vaginal | <2500 | 8-10 | NO SEPSIS |
| Early-onset sepsis | 2-4 | 26-35 | >4 | 1-3 | >/-=37 | Male | No | Caesarean section | >/=2500 | 5-7 | SEPSIS |
| Other illnesses | >/=5 | 26-35 | >4 | 1-3 | >/-=37 | Female | No | Vaginal | <2500 | 8-10 | NO SEPSIS |
| Early-onset sepsis | 2-4 | 26-35 | >4 | 1-3 | >/-=37 | Male | No | Vaginal | >/=2500 | 5-7 | SEPSIS |
| Other illnesses | >/=5 | >35 | 1-4 | 1-3 | <37 | Female | No | Caesarean section | <2500 | 8-10 | NO SEPSIS |
| Other illnesses | 1 | <20 | 1-4 | 1-3 | <37 | Female | Yes | Caesarean section | <2500 | 8-10 | NO SEPSIS |
| Early-onset sepsis | 2-4 | 26-35 | 1-4 | 1-3 | >/-=37 | Female | Yes | Vaginal | >/=2500 | 8-10 | SEPSIS |
| Other illnesses | 2-4 | 20-25 | 1-4 | 1-3 | <37 | Male | Yes | Vaginal | <2500 | 8-10 | NO SEPSIS |
| Early-onset sepsis | 1 | <20 | >4 | 1-3 | >/-=37 | Female | Yes | Caesarean section | >/=2500 | 1-4 | SEPSIS |
| Late-onset sepsis | 2-4 | 26-35 | >4 | 4-28 | <37 | Male | Yes | Vaginal | <2500 | 8-10 | SEPSIS |
| Other illnesses | >/=5 | 26-35 | >4 | 4-28 | <37 | Female | No | Vaginal | <2500 | 1-4 | NO SEPSIS |
| Early-onset sepsis | 2-4 | 20-25 | >4 | 1-3 | <37 | Female | Yes | Caesarean section | <2500 | 5-7 | SEPSIS |
| Early-onset sepsis | 2-4 | 20-25 | >4 | 4-28 | <37 | Male | No | Vaginal | <2500 | 8-10 | SEPSIS |
| Other illnesses | 2-4 | 20-25 | >4 | 1-3 | >/-=37 | Female | No | Vaginal | >/=2500 | 5-7 | NO SEPSIS |
| Early-onset sepsis | 2-4 | 20-25 | 1-4 | 1-3 | <37 | Male | Yes | Vaginal | <2500 | 5-7 | SEPSIS |
| Other illnesses | 2-4 | 20-25 | 1-4 | 1-3 | <37 | Male | No | Vaginal | <2500 | 5-7 | NO SEPSIS |
| Early-onset sepsis | 2-4 | 20-25 | 1-4 | 1-3 | >/-=37 | Female | Yes | Vaginal | >/=2500 | 1-4 | SEPSIS |
| Other illnesses | 2-4 | 26-35 | >4 | 1-3 | >/-=37 | Male | Yes | Vaginal | <2500 | 8-10 | NO SEPSIS |
| Other illnesses | 1 | <20 | >4 | 1-3 | >/-=37 | Female | Yes | Caesarean section | >/=2500 | 8-10 | NO SEPSIS |
| Early-onset sepsis | >/=5 | 26-35 | 1-4 | 1-3 | >/-=37 | Male | No | Caesarean section | >/=2500 | 5-7 | SEPSIS |
| Other illnesses | 2-4 | 20-25 | >4 | 1-3 | <37 | Male | Yes | Caesarean section | <2500 | 8-10 | NO SEPSIS |
| Other illnesses | 2-4 | 26-35 | >4 | 1-3 | <37 | Female | No | Caesarean section | <2500 | 8-10 | NO SEPSIS |
| Necrotizing enterocolitis | >/=5 | >35 | 1-4 | 4-28 | >/-=37 | Male | Yes | Assisted vaginal | >/=2500 | 8-10 | SEPSIS |
| Necrotizing enterocolitis | 2-4 | 20-25 | 1-4 | 4-28 | >/-=37 | Female | Yes | Vaginal | <2500 | 5-7 | SEPSIS |
| Other illnesses | 2-4 | 20-25 | >4 | 1-3 | >/-=37 | Female | No | Vaginal | <2500 | 8-10 | NO SEPSIS |
| Other illnesses | 1 | 26-35 | >4 | 1-3 | >/-=37 | Male | No | Caesarean section | <2500 | 5-7 | NO SEPSIS |
| Necrotizing enterocolitis | 1 | 20-25 | >4 | 1-3 | >/-=37 | Male | Yes | Caesarean section | >/=2500 | 5-7 | SEPSIS |
| Other illnesses | 2-4 | 26-35 | 1-4 | 1-3 | <37 | Female | Yes | Caesarean section | <2500 | 5-7 | NO SEPSIS |
| Other illnesses | 1 | <20 | 1-4 | 4-28 | <37 | Male | Yes | Vaginal | <2500 | 5-7 | NO SEPSIS |
| presumed sepsis | 2-4 | 20-25 | >4 | 1-3 | >/-=37 | Male | Yes | Vaginal | >/=2500 | 5-7 | SEPSIS |
| Other illnesses | 2-4 | 20-25 | >4 | 1-3 | >/-=37 | Female | Yes | Caesarean section | >/=2500 | 5-7 | NO SEPSIS |
| presumed sepsis | 2-4 | 20-25 | >4 | 1-3 | >/-=37 | Female | Yes | Caesarean section | >/=2500 | 5-7 | SEPSIS |
| Necrotizing enterocolitis | 2-4 | 20-25 | 1-4 | 4-28 | <37 | Male | Yes | Vaginal | <2500 | 5-7 | SEPSIS |
| Early-onset sepsis | 2-4 | 26-35 | >4 | 1-3 | >/-=37 | Female | Yes | Assisted vaginal | >/=2500 | 8-10 | SEPSIS |
| Other illnesses | 1 | <20 | >4 | 1-3 | <37 | Female | Yes | Caesarean section | <2500 | 8-10 | NO SEPSIS |
| Other illnesses | 2-4 | 26-35 | 1-4 | 1-3 | <37 | Male | Yes | Assisted vaginal | <2500 | 8-10 | NO SEPSIS |
| Late-onset sepsis | 1 | 20-25 | 1-4 | 4-28 | <37 | Female | No | Vaginal | <2500 | 8-10 | SEPSIS |
| Other illnesses | 2-4 | 26-35 | >4 | 1-3 | >/-=37 | Male | No | Vaginal | <2500 | 8-10 | NO SEPSIS |
| Early-onset sepsis | 1 | 20-25 | >4 | 1-3 | <37 | Female | Yes | Vaginal | <2500 | 8-10 | SEPSIS |
| Late-onset sepsis | 2-4 | 20-25 | >4 | 4-28 | >/-=37 | Female | Yes | Vaginal | >/=2500 | 8-10 | SEPSIS |
| Late-onset sepsis | 2-4 | 26-35 | >4 | 4-28 | >/-=37 | Male | No | Vaginal | >/=2500 | 8-10 | SEPSIS |
| Other illnesses | 1 | <20 | 1-4 | 1-3 | >/-=37 | Male | Yes | Vaginal | >/=2500 | 8-10 | NO SEPSIS |
| Early-onset sepsis | 2-4 | 20-25 | >4 | 1-3 | >/-=37 | Male | No | Vaginal | >/=2500 | 5-7 | SEPSIS |
| Other illnesses | 2-4 | 20-25 | 1-4 | 1-3 | <37 | Male | Yes | Vaginal | <2500 | 8-10 | NO SEPSIS |
| Other illnesses | 1 | <20 | 1-4 | 1-3 | <37 | Male | Yes | Vaginal | <2500 | 8-10 | NO SEPSIS |
| Other illnesses | >/=5 | >35 | >4 | 1-3 | >/-=37 | Female | Yes | Vaginal | >/=2500 | 5-7 | NO SEPSIS |
| Other illnesses | 2-4 | 26-35 | 1-4 | 1-3 | <37 | Female | Yes | Vaginal | <2500 | 5-7 | NO SEPSIS |
| Other illnesses | 1 | 20-25 | >4 | 4-28 | <37 | Female | Yes | Caesarean section | >/=2500 | 5-7 | NO SEPSIS |
| Late-onset sepsis | 1 | 20-25 | >4 | 4-28 | <37 | Male | No | Vaginal | <2500 | 5-7 | SEPSIS |
| Other illnesses | 1 | 20-25 | >4 | 1-3 | <37 | Male | Yes | Caesarean section | <2500 | 5-7 | NO SEPSIS |
| Other illnesses | 2-4 | <20 | 1-4 | 1-3 | <37 | Female | Yes | Caesarean section | >/=2500 | 5-7 | NO SEPSIS |
| Other illnesses | 1 | 20-25 | >4 | 1-3 | <37 | Female | No | Vaginal | <2500 | 5-7 | NO SEPSIS |
| Late-onset sepsis | 2-4 | 20-25 | 1-4 | 4-28 | >/-=37 | Female | No | Vaginal | >/=2500 | 5-7 | SEPSIS |
| Early-onset sepsis | 2-4 | 20-25 | >4 | 1-3 | >/-=37 | Female | No | Vaginal | >/=2500 | 1-4 | SEPSIS |
| Other illnesses | 2-4 | 26-35 | 1-4 | 1-3 | <37 | Female | Yes | Vaginal | <2500 | 8-10 | NO SEPSIS |
| Other illnesses | 2-4 | 20-25 | 1-4 | 1-3 | <37 | Female | Yes | Caesarean section | <2500 | 8-10 | NO SEPSIS |
| Other illnesses | 2-4 | 20-25 | >4 | 1-3 | <37 | Male | No | Vaginal | <2500 | 8-10 | NO SEPSIS |
| Early-onset sepsis | 1 | >35 | >4 | 1-3 | >/-=37 | Female | Yes | Vaginal | >/=2500 | 8-10 | SEPSIS |
| Other illnesses | 1 | <20 | >4 | 1-3 | <37 | Female | No | Vaginal | <2500 | 8-10 | NO SEPSIS |
| Staphylococcal skin infection | 1 | 20-25 | >4 | 4-28 | >/-=37 | Male | No | Vaginal | >/=2500 | 8-10 | SEPSIS |
| Other illnesses | 1 | <20 | >4 | 1-3 | >/-=37 | Male | Yes | Vaginal | <2500 | 5-7 | NO SEPSIS |
| Early-onset sepsis | 2-4 | 20-25 | 1-4 | 1-3 | <37 | Female | Yes | Vaginal | <2500 | 8-10 | SEPSIS |
| Early-onset sepsis | 1 | 20-25 | >4 | 1-3 | >/-=37 | Male | Yes | Vaginal | >/=2500 | 5-7 | SEPSIS |
| Other illnesses | 1 | 20-25 | >4 | 1-3 | >/-=37 | Female | Yes | Vaginal | >/=2500 | 8-10 | NO SEPSIS |
| Other illnesses | >/=5 | 26-35 | 1-4 | 1-3 | <37 | Male | No | Vaginal | <2500 | 5-7 | NO SEPSIS |
| Early-onset sepsis | 2-4 | 20-25 | >4 | 1-3 | <37 | Male | Yes | Vaginal | <2500 | 8-10 | SEPSIS |
| Other illnesses | 2-4 | 20-25 | >4 | 1-3 | <37 | Male | No | Vaginal | <2500 | 8-10 | NO SEPSIS |
| Other illnesses | 2-4 | 26-35 | 1-4 | 1-3 | <37 | Female | No | Vaginal | <2500 | 8-10 | NO SEPSIS |
| presumed sepsis | 2-4 | 20-25 | >4 | 1-3 | <37 | Male | Yes | Vaginal | <2500 | 8-10 | SEPSIS |
| Early-onset sepsis | 2-4 | 20-25 | >4 | 1-3 | >/-=37 | Male | Yes | Vaginal | >/=2500 | 8-10 | SEPSIS |
| Other illnesses | 1 | <20 | >4 | 1-3 | <37 | Female | Yes | Vaginal | >/=2500 | 8-10 | NO SEPSIS |
| Other illnesses | 1 | <20 | >4 | 1-3 | >/-=37 | Male | Yes | Vaginal | <2500 | 8-10 | NO SEPSIS |
| Early-onset sepsis | 1 | <20 | >4 | 1-3 | >/-=37 | Female | Yes | Vaginal | >/=2500 | 5-7 | SEPSIS |
| Other illnesses | 1 | <20 | >4 | 1-3 | >/-=37 | Male | Yes | Caesarean section | >/=2500 | 5-7 | NO SEPSIS |
| Other illnesses | 1 | 20-25 | >4 | 1-3 | <37 | Male | Yes | Vaginal | <2500 | 1-4 | NO SEPSIS |
| Other illnesses | 2-4 | 20-25 | >4 | 1-3 | >/-=37 | Male | No | Vaginal | >/=2500 | 5-7 | NO SEPSIS |
| Early-onset sepsis | 1 | 20-25 | >4 | 1-3 | >/-=37 | Male | Yes | Vaginal | >/=2500 | 1-4 | SEPSIS |
| Other illnesses | 2-4 | 26-35 | >4 | 1-3 | >/-=37 | Male | Yes | Caesarean section | >/=2500 | 5-7 | NO SEPSIS |
| Other illnesses | 2-4 | 20-25 | >4 | 4-28 | <37 | Female | Yes | Vaginal | >/=2500 | 5-7 | NO SEPSIS |
| Other illnesses | 1 | 20-25 | >4 | 1-3 | >/-=37 | Male | Yes | Vaginal | >/=2500 | 5-7 | NO SEPSIS |
| Early-onset sepsis | >/=5 | >35 | 1-4 | 4-28 | >/-=37 | Female | Yes | Vaginal | >/=2500 | 1-4 | SEPSIS |
| Necrotizing enterocolitis | 1 | <20 | >4 | 1-3 | >/-=37 | Female | No | Vaginal | >/=2500 | 1-4 | SEPSIS |
| Other illnesses | 1 | <20 | >4 | 1-3 | >/-=37 | Male | No | Vaginal | >/=2500 | 1-4 | NO SEPSIS |
| Other illnesses | 1 | 26-35 | >4 | 4-28 | <37 | Male | No | Vaginal | <2500 | 8-10 | NO SEPSIS |
| Other illnesses | 2-4 | 26-35 | 1-4 | 1-3 | >/-=37 | Male | No | Caesarean section | >/=2500 | 8-10 | NO SEPSIS |
| Other illnesses | 2-4 | 26-35 | >4 | 1-3 | >/-=37 | Male | Yes | Vaginal | >/=2500 | 8-10 | NO SEPSIS |
| Other illnesses | 1 | 20-25 | 1-4 | 1-3 | <37 | Male | Yes | Vaginal | <2500 | 8-10 | NO SEPSIS |
| Early-onset sepsis | 1 | 20-25 | 1-4 | 1-3 | <37 | Female | Yes | Vaginal | <2500 | 8-10 | SEPSIS |
| Early-onset sepsis | 2-4 | 20-25 | >4 | 1-3 | <37 | Female | No | Vaginal | <2500 | 8-10 | SEPSIS |
| Early-onset sepsis | 1 | 20-25 | 1-4 | 1-3 | >/-=37 | Male | Yes | Vaginal | <2500 | 8-10 | SEPSIS |
| Early-onset sepsis | 1 | <20 | >4 | 4-28 | <37 | Male | Yes | Assisted vaginal | <2500 | 8-10 | SEPSIS |
| Other illnesses | 1 | <20 | >4 | 4-28 | <37 | Male | No | Vaginal | <2500 | 1-4 | NO SEPSIS |
| Other illnesses | 1 | 20-25 | 1-4 | 1-3 | <37 | Male | No | Vaginal | >/=2500 | 5-7 | NO SEPSIS |
| Early-onset sepsis | 2-4 | 20-25 | 1-4 | 1-3 | <37 | Female | No | Vaginal | <2500 | 5-7 | SEPSIS |
| Other illnesses | >/=5 | >35 | 1-4 | 1-3 | <37 | Male | Yes | Vaginal | <2500 | 8-10 | NO SEPSIS |
| Other illnesses | 2-4 | 20-25 | >4 | 1-3 | <37 | Male | No | Vaginal | <2500 | 8-10 | NO SEPSIS |
| Necrotizing enterocolitis | 1 | <20 | >4 | 1-3 | <37 | Female | No | Caesarean section | <2500 | 5-7 | SEPSIS |
| Other illnesses | 2-4 | 20-25 | 1-4 | 1-3 | <37 | Female | No | Vaginal | <2500 | 8-10 | NO SEPSIS |
| Early-onset sepsis | 1 | 20-25 | >4 | 1-3 | >/-=37 | Male | Yes | Vaginal | >/=2500 | 8-10 | SEPSIS |
| Other illnesses | 2-4 | 20-25 | >4 | 4-28 | >/-=37 | Male | Yes | Caesarean section | >/=2500 | 8-10 | NO SEPSIS |
| Early-onset sepsis | 2-4 | 20-25 | 1-4 | 1-3 | >/-=37 | Female | Yes | Vaginal | >/=2500 | 8-10 | SEPSIS |
| Other illnesses | 2-4 | 26-35 | >4 | 1-3 | >/-=37 | Male | Yes | Vaginal | >/=2500 | 8-10 | NO SEPSIS |
| Meningitis | 1 | 20-25 | >4 | 1-3 | >/-=37 | Female | No | Caesarean section | >/=2500 | 8-10 | SEPSIS |
| Other illnesses | 1 | 20-25 | >4 | 1-3 | <37 | Male | No | Caesarean section | <2500 | 8-10 | NO SEPSIS |
| Other illnesses | 1 | <20 | >4 | 1-3 | <37 | Male | No | Vaginal | <2500 | 8-10 | NO SEPSIS |
| Other illnesses | 1 | 20-25 | >4 | 1-3 | <37 | Male | No | Vaginal | <2500 | 5-7 | NO SEPSIS |
| Early-onset sepsis | 2-4 | 20-25 | 1-4 | 1-3 | >/-=37 | Female | Yes | Vaginal | >/=2500 | 8-10 | SEPSIS |
| presumed sepsis | 2-4 | 20-25 | >4 | 1-3 | >/-=37 | Female | Yes | Caesarean section | >/=2500 | 1-4 | SEPSIS |
| Other illnesses | 2-4 | 26-35 | 1-4 | 1-3 | >/-=37 | Female | Yes | Vaginal | >/=2500 | 8-10 | NO SEPSIS |
| Other illnesses | >/=5 | >35 | >4 | 1-3 | >/-=37 | Male | Yes | Vaginal | >/=2500 | 1-4 | NO SEPSIS |
| Other illnesses | 2-4 | 20-25 | 1-4 | 1-3 | <37 | Female | No | Caesarean section | <2500 | 8-10 | NO SEPSIS |
| Other illnesses | 1 | 20-25 | >4 | 1-3 | >/-=37 | Male | Yes | Caesarean section | >/=2500 | 8-10 | NO SEPSIS |
| Other illnesses | >/=5 | 26-35 | 1-4 | 1-3 | <37 | Female | No | Vaginal | <2500 | 8-10 | NO SEPSIS |
| Other illnesses | 2-4 | 20-25 | >4 | 1-3 | >/-=37 | Female | Yes | Caesarean section | >/=2500 | 8-10 | NO SEPSIS |
| Other illnesses | 1 | <20 | 1-4 | 1-3 | <37 | Male | No | Vaginal | <2500 | 8-10 | NO SEPSIS |
| Other illnesses | >/=5 | 26-35 | 1-4 | 1-3 | >/-=37 | Male | Yes | Caesarean section | >/=2500 | 8-10 | NO SEPSIS |
| Other illnesses | 2-4 | 26-35 | >4 | 1-3 | <37 | Female | No | Vaginal | <2500 | 8-10 | NO SEPSIS |
| Other illnesses | 1 | 20-25 | 1-4 | 1-3 | <37 | Male | No | Caesarean section | <2500 | 8-10 | NO SEPSIS |
| Other illnesses | 1 | <20 | 1-4 | 4-28 | >/-=37 | Male | Yes | Vaginal | >/=2500 | 8-10 | NO SEPSIS |
| Other illnesses | 1 | <20 | >4 | 1-3 | <37 | Male | Yes | Vaginal | <2500 | 5-7 | NO SEPSIS |
| Other illnesses | 2-4 | 20-25 | 1-4 | 1-3 | <37 | Female | No | Vaginal | >/=2500 | 5-7 | NO SEPSIS |
| Other illnesses | 1 | <20 | >4 | 1-3 | <37 | Male | No | Vaginal | <2500 | 8-10 | NO SEPSIS |
| Other illnesses | 2-4 | 26-35 | >4 | 1-3 | <37 | Female | Yes | Caesarean section | <2500 | 5-7 | NO SEPSIS |
| Other illnesses | 1 | <20 | 1-4 | 1-3 | <37 | Female | Yes | Caesarean section | <2500 | 8-10 | NO SEPSIS |
| Other illnesses | 1 | 20-25 | >4 | 1-3 | >/-=37 | Female | Yes | Vaginal | >/=2500 | 8-10 | NO SEPSIS |
| Early-onset sepsis | 1 | <20 | 1-4 | 1-3 | >/-=37 | Male | Yes | Vaginal | >/=2500 | 8-10 | SEPSIS |
| Other illnesses | 1 | 20-25 | >4 | 1-3 | >/-=37 | Male | Yes | Vaginal | >/=2500 | 5-7 | NO SEPSIS |
| Early-onset sepsis | 1 | <20 | >4 | 1-3 | >/-=37 | Female | Yes | Vaginal | >/=2500 | 1-4 | SEPSIS |
| Other illnesses | >/=5 | >35 | 1-4 | 1-3 | >/-=37 | Male | Yes | Caesarean section | <2500 | 8-10 | NO SEPSIS |
| Other illnesses | 2-4 | 20-25 | 1-4 | 1-3 | >/-=37 | Female | No | Caesarean section | <2500 | 5-7 | NO SEPSIS |
| Other illnesses | 1 | <20 | 1-4 | 1-3 | <37 | Male | No | Vaginal | <2500 | 1-4 | NO SEPSIS |
| Early-onset sepsis | 2-4 | 20-25 | 1-4 | 1-3 | <37 | Female | Yes | Vaginal | >/=2500 | 5-7 | SEPSIS |
| Other illnesses | 2-4 | 20-25 | 1-4 | 1-3 | >/-=37 | Female | No | Vaginal | <2500 | 5-7 | NO SEPSIS |
| Other illnesses | >/=5 | 26-35 | >4 | 1-3 | <37 | Female | No | Vaginal | <2500 | 8-10 | NO SEPSIS |
| Other illnesses | 1 | <20 | 1-4 | 1-3 | >/-=37 | Female | Yes | Caesarean section | >/=2500 | 8-10 | NO SEPSIS |
| Other illnesses | 1 | <20 | 1-4 | 1-3 | <37 | Female | No | Vaginal | <2500 | 8-10 | NO SEPSIS |
