# Supplementary material for: Prevalence of neonatal sepsis and associated factors among neonates admitted in the neonatal intensive care unit at Lira Regional Referral Hospital, Northern Uganda
Source: PLoS One. 2025 Jan 13;20(1):e0315794. doi: 10.1371/journal.pone.0315794 (PMC11730428; doi:10.1371/journal.pone.0315794)
Supplement: Attachment — Submitted filename: RESPONSE TO REVIEWER.docx [file pone.0315794.s002.docx]

BRENDAH KATUGUME

0761251221/0741694346

[brendahkatugume2000@gmail.com](mailto:brendahkatugume2000@gmail.com)

Kyegegwa Uganda.

30/01/2024.

RE: RESPONSE TO REVIEWERS.

Dear Sir/madam,

I hereby write to present my gratitude to you for the valuable comments towards improving the research paper. We are sorry about the mistakes in our manuscript. According to the comments we have attached the revised version of the manuscript and hope that these revisions and the improved text will be satisfactory.

| Journal requirements | My responses |
| --- | --- |
| 1. Please ensure that your manuscript meets PLOS ONE's style requirements, including those for file naming. The PLOS ONE style templates can be found at  <https://journals.plos.org/plosone/s/file?id=wjVg/PLOSOne_formatting_sample_main_body.pdf> and  <https://journals.plos.org/plosone/s/file?id=ba62/PLOSOne_formatting_sample_title_authors_affiliations.pdf> | The steps to submission were reviewed and this was addressed. |
| 2. Thank you for stating the following in the Acknowledgments Section of your manuscript:  "I would like to acknowledge the research supervisor Ms. Namutebi Deborah Andrinar for her unfailing effort to the success of the study. My sincere appreciation to my beloved research lecturers at Lira university Mr. Udho Samson, Dr Amir Kabunga and Dr Rita Matte.  I am grateful to Dr. Nelson Okello and Dr. John Baptist Muzungu for the helping hand rendered in development of the data extraction tool. Am grateful to the department of midwifery, Faculty of Nursing and Midwifery and Lira University at large, LUREC, staff of NICU of LRRH and the whole administration of the hospital for enabling the study to be conducted. Furthermore, I am grateful to Mr. Eustes Kigongo for helping me understand and do data entry and analysis, friends Namawuba Vanessa Ruth and Ssuuna Thomasmoore for encouraging me and finally acknowledge my mother Ms. Kabatooro Rosemary for the unfailing financial support to the success of the study."  We note that you have provided funding information that is not currently declared in your Funding Statement. However, funding information should not appear in the Acknowledgments section or other areas of your manuscript. We will only publish funding information present in the Funding Statement section of the online submission form.  Please remove any funding-related text from the manuscript and let us know how you would like to update your Funding Statement. Currently, your Funding Statement reads as follows:  "The author(s) received no specific funding for this work."  Please include your amended statements within your cover letter; we will change the online submission form on your behalf. | The funding statement that had been included was removed since the funds were not from any organization but just a guardian therefore the financial statement still stands that no specific funds were acquired. |
| 3. In your Data Availability statement, you have not specified where the minimal data set underlying the results described in your manuscript can be found. PLOS defines a study's minimal data set as the underlying data used to reach the conclusions drawn in the manuscript and any additional data required to replicate the reported study findings in their entirety. All PLOS journals require that the minimal data set be made fully available. For more information about our data policy, please see <http://journals.plos.org/plosone/s/data-availability>.  Upon re-submitting your revised manuscript, please upload your study’s minimal underlying data set as either Supporting Information files or to a stable, public repository and include the relevant URLs, DOIs, or accession numbers within your revised cover letter. For a list of acceptable repositories, please see <http://journals.plos.org/plosone/s/data-availability#loc-recommended-repositories>. Any potentially identifying patient information must be fully anonymized.  Important: If there are ethical or legal restrictions to sharing your data publicly, please explain these restrictions in detail. Please see our guidelines for more information on what we consider unacceptable restrictions to publicly sharing data: <http://journals.plos.org/plosone/s/data-availability#loc-unacceptable-data-access-restrictions>. Note that it is not acceptable for the authors to be the sole named individuals responsible for ensuring data access.  We will update your Data Availability statement to reflect the information you provide in your cover letter. | The minimal data set that was used to reach the conclusion has been attached as supporting information. |
| 4. We notice that your supplementary figures are uploaded with the file type 'Figure'. Please amend the file type to 'Supporting Information'. Please ensure that each Supporting Information file has a legend listed in the manuscript after the references list. | This was amended |
| 5. Please upload a copy of Figure 1, to which you refer in your text on page 10. If the figure is no longer to be included as part of the submission please remove all reference to it within the text. | This has been addressed |
| Reviewer #1: I would like to thank the authors for investigating such an important topic that is relevant to clinical practice and the improvement of neonatal health. Below are comments regarding the manuscript:  General concerns The study is good; however, some major review is needed to improve it. The work needs editing to improve the sentence construction and typing errors The whole text needs sentence numbering, this will make review and comments easy  Abstract Introduction: Could there be statistics for sub-Saharan Africa other than relating your study to Global estimates? Global estimates fit the background section in the main text. Your objective talks about the Lango sub-region; however, your study was carried out at Lira Regional Referral Hospital. Can the result represent the sub-region? Methods I think “A hospital-based cross-sectional study with a retrospective chart review was conducted….” Leaving out quantitative The sentences are too long. This concerns the whole manuscript. Try to shorten them. Also, you need not begin a sentence with “using” Results: Make the adjusted odds ratio (AOR) and confidence interval (CI) uniform across all the variables presented Conclusion The recommendation is broad. Are you saying all newborns should receive prophylaxis?  Background This section is quite long. I would suggest you reduce the text. Kindly number the sentences. This can be done from the Word document (layout). Keep the sentences short, simple and clear. The whole of the third paragraph is not cited. Also, the last 4 lines of this paragraph can be removed The last 5 lines of the last paragraph are not for this section, take it to the method section.  Methods and materials Study area Any idea, how many babies are managed in the NICU annually Population Inclusion and exclusion criteria are missing Ethics approval and consent to participate You may need to edit the paragraph. Remove some details that are not very necessary. You may consult articles for guidance. Data collection and quality control This section has no information on the data collection procedure. It is mainly a description of the research instrument. Please explain how the data were collected. Also, you may need to define the variables considered and how they were measured. For instance, how was the gestation age determined? Also, how did you arrive at the diagnosis of neonatal sepsis? Data processing and analysis This paragraph also needs editing. Some sentences are not necessary e.g. data analysis was done at…. “Bivariate analysis was performed using…….” This sentence is not clear Also, when you say “bivariate” what do you mean? I guess you mean bivariable The backward elimination method was performed….. when was this done and why? Confounding and interactions were assessed………. What was the result of your assessment? How did you control for confounding?  Results This section also needs editing and removing some paragraphs. You may consider being brief and straight to the point. In the results, readers want to see a) how many charts (neonates) provided information for this study? Or the number of neonatal information/records reviewed. What were the proportions of your outcome (neonatal sepsis) and other conditions if available, and descriptions of the neonates and maternal (characteristics)? A piece of brief information is explained in a narrative while details are included in a table b) factors associated with neonatal sepsis. Here you pick out variables that were significant while including their adjusted Odds ratio and confidence interval. No need to dedicate a whole paragraph to bivariable analysis. This can remain on the table. You can construct a table with both crude and adjusted odds ratios, p values etc. Change title from maternal factors to maternal characteristics The title “bivariate…… of factors…., makes it factors associated with neonatal sepsis, indicated in (b) above Tables: please put the title at the top of the table, and align figures with the variable name. For instance, Table 4: factors associated with neonatal sepsis among neonates admitted at NICU, LRRH. Edit all the tables. In the text, you may not include P values. The readers can see from the table. Check the variable names in Table 5 Kindly include variable names and their description in the method section so that one can make meaning of your findings. Generally, improve on the presentation of your results. You may consult some articles for guidance. Please, present the outcome of both bivariable and multivariable analysis in one table, if possible, with crude and adjusted OR and the P value of the adjusted OR. Discussion It's advisable not to discuss or explain other people’s studies while relating to your study. You have not mentioned how the sepsis was diagnosed in the methods. Now you have included it in the discussion. Your discussion mainly has studies from Ethiopia, do you mean studies conducted in Uganda are scarce? Here you mention low birth weight. According to your data. Who are they? It is common to find facts with no citation in this section. Kindly cite information that is not yours. What were the strengths and limitations of this study? A conclusion is absent. You need to include it.  Figure You may need to use comparative colours to give a difference in the pie chart. (Sepsis and no sepsis). Make one lighter and another one darker. The figure should have a label below Remove the title inside the figure | These have been addressed  The global statistics have been replaced with those of sub-Sahara Africa.  This has been addressed in the cover letter attached.  These have been addressed in the manuscript.  This has been addressed.  This was eliminated and an adjusted table added.  This has been summarized.  This has been addressed.  These will be added, it was delayed by the records office but it will be added.  These have been added.  This has been added  This has been addressed in the manuscript.  This was addressed  These were addressed.  This was addressed  This has been addressed.  This has been addressed  This has been addressed.  This has been addressed.  This has been addressed in the cover letter  This has been addressed.  These have been added.  This has been addressed |
| Reviewer #2: Thanks you for reviewing your articles entitled Prevalence of neonatal sepsis and associated factors among neonates admitted in the neonatal intensive care unit at Lira Regional Referral Hospital, northern Uganda. I have some comments and suggestions  Abstract part * It says 80% have neonatal sepsis. At the same time it says 41%. So which one is correct? * according to this study the APGAR score of 1-4 is protective.. how low APGAR score become protective raises the questions on the validity of the study. In this case it need resuscitation and that may be risk for the sepsis as many evidence suggested. Why your study is differ is not clear. * Over all the result part need to rewrite. Introduction  Need to use more updated data. . Justification for conducted this particular was not acceptable Methods and Materials 1. The research lack study population, inclusion and Exclusion Criteria. 2. Ethical consideration is also other issues. 2.1 it should be after data processing and analysis. 2.3 it should be short and to the point. 2.3. There is no any verb indicated you did not collect personal identifiers. 3 Sample size, method and techniques 3.1 Sample size calculation is not clear. I doubt its scientific. 3.2 Why you didn't used simple random sampling as your study was retrospective? 3.3 you said "198 respondents record " did you really have respondents? 3.4 which number was sellected from 1 to 2 in the first. 3.5 it say 198 and 194 so which one is the correct one? 4. Data collection process 4.1 Who were your data collectors 4.2 why you prefer UPA tool than any other standard tools. 4.3 how you keep quality of your research 4.4. How you measured your outcome variable 4.5 there are numerous problems in grammar and the chronological order to scientific papers. It look like the short note writing. 4.6 there is no dependent and independent variables. And how to measure outcomes variables was missed from the study. 4.7 ita said data collection tools taken from UPA and it also said. Quality was controlled by using other study tools. Please which one is correct? 4.8 do you think this is enough to control your the quality of your research. 5. Data analysis methods 5.1 Need to rewrite. Because it says *(p-value <0.20 used to get candidates for multivariate analysis. Again it say backwards stepwise methods was used" I confused with such analysis?.. I think it needs to rewrite or to stick either of the two. 5.2. How you control confound and interaction should be reported. 5.2 overall it need to rewrite again. 6.1 Repetition of idea in the first 7 line of the result should be summerized. 6.2 majority of neonates were low birth weight could you give us justification ? 6.3 many important variables of the maternal missed 6.4 in the table APGAR 8_10 ---no observation reported, why? 6.7.if you observe your cross tab indicates most of neonates with normal birth, with resuscitation , low APGAR score have high sepsis prevalence. Why??? 6.8 check APGAR score it say April......what you mean that?? 6.8. Many variables missed 6.8. Merge bivariate and multivariate analysis. 6.9 I have doubt that the method of data analy was no well implemented 7. Discussion Not well organised 8. The research lacks many important components: strengths, limitations, conclusion and recommendations . | This has been addressed it was a typing error.  This has been addressed.  These have been included in the manuscript.  This has been addressed.  A statement has been added to the manuscript.  The formular was added.  Even simple systematic sampling is also considered for retrospective studies.  This was addressed.  This has been addressed.  This was addressed.  This was addressed.  These were labelled.  This was addressed.  This was addressed ad oe of the confusing statements elimitted.  This was addressed.  This was addressed.  This was edited and pointed to all neonates at risk.  This was addressed.  These were edited.  This was addressed.  These were added. |
