# Supplementary material for: Prevalence of neonatal sepsis and associated factors among neonates admitted in the neonatal intensive care unit at Lira Regional Referral Hospital, Northern Uganda
Source: PLoS One. 2025 Jan 13;20(1):e0315794. doi: 10.1371/journal.pone.0315794 (PMC11730428; doi:10.1371/journal.pone.0315794)
Supplement: Attachment — Submitted filename: Response to reviewers..docx [file pone.0315794.s003.docx]

BRENDAH KATUGUME

+256 761 251 221/ 741 694 346

[brendahkatugume2000@gmail.com](mailto:brendahkatugume2000@gmail.com)

Kyegegwa Uganda.

19/04/2024.

TO THE ACADEMIC EDITOR,

PLOS ONE JOURNAL

Dear Dr.

RE: **RESPONSE TO REVIEWER COMMENTS.**

I hereby write to appreciate you doctor and the journal at large for considering our work for review, the reviewers as well for giving us very important comments aimed at improving the quality of our work.

This letter is to inform you that I have duly received and appreciated the manuscript evaluation report of the study entitled **Prevalence of neonatal sepsis and associated factors among neonates admitted to NICU of LRRH** given on 7th of March 2024 at 05:13 Am. Although I agree with some of the evaluations, I shall respectfully make some reservations.

| S/NO | REVIEWER’S COMMENT | AUTHOR’S RESPONSE | PAGE NUMBER |
| --- | --- | --- | --- |
| 1. | Neonatal sepsis is a leading cause of neonatal morbidity and mortality in the low resource settings. The concept was well though and the importance of the area of study cannot be underestimated. The prevalence was alarmingly high! | Thank you for commending our work. |  |
| 2. | There’s generally a need to correct the numerous typographical and grammatical errors much as these errors do not adversely affect the results | Thank you for considering our work, the grammar of the document has been corrected as indicated in the revised manuscript with tracked changes. | The entire document. |
| 3. | Also, be consistent in the line spacing and font type and size! | Thank you so much, this too has been addressed. | The whole document. |
| 4. | Abstract: You mention that the study was done in the Lango sub-region. Is Lira hospital, which was the epicentre of the study, representative of the Lango sub-region? If not, please state exactly where the study was done i.e Lira regional referral hospital! | Am glad about the comment. the study area was edited and instead of lango subregion it was narrowed back to Lira Regional Referral Hospital with an explanation of the study being conducted in LRRH only | 1,2. |
| 5. | Introduction: The introduction was well written and contains relevant information to the study. There is however a mix in the referencing/citation style. Please stick to one referencing style and be consistent | Thank you so much for pointing this out. The point of mixed citations was because citation was done with different referencing managers but that was edited and redone using the sme referencing manager through out the whole manuscript. | 3,4. |
| 6. | Methods and materials: There’s need to mention or write a sentence or two on the capacity of Lira Hospital in regard to neonatal care services and the NICU. This will give the reader an insight into the justification for the study at that site. | Am glad that i have been reminded about this, it too has been addressed. The capacity of the NICU was also included and that considered 2 years, 2021 and 2022. | 5 |
| 7. | The rest of the methods section is easy to follow through! | Thank you for commending our work |  |
| 8. | Results: The results are informative. | We are grateful about the comment. |  |
| 9. | Table 5 on factors associated with neonatal sepsis needs to be complete by including all the p-values and ORs for the variables listed! | Thank you for the comment. For this comment, no editing has been done to the table but the explanation has been provided. Part of the odds ratios of table 5 are missing because those particular variables were not considered for multivariable analysis since they had a p-value that was bigger than 0.2 which was significant for that level in data analysis. | 14 |
| 10. | Discussion: There’s some attempt to explain the comparisons between the study and studies cited | Thank you for commending our work. |  |
| 11. | The recommendations need to be directed to entities that the author (s) believe will be able to drive the recommendation. | Thank you the comments. The point of having the recommendations being directed has been done too as per the tracked changes. | 17 |

I will be grateful if my views, explanations and reservations are put under positive considerations.

Yours faithfully,

BRENDAH KATUGUME.

PRINCIPAL INVESTIGATOR.
