# Supplementary material for: Prevalence of neonatal sepsis and associated factors among neonates admitted in the neonatal intensive care unit at Lira Regional Referral Hospital, Northern Uganda
Source: PLoS One. 2025 Jan 13;20(1):e0315794. doi: 10.1371/journal.pone.0315794 (PMC11730428; doi:10.1371/journal.pone.0315794)
Supplement: Attachment — Submitted filename: RESPONSE TO REVIEWER new.docx [file pone.0315794.s004.docx]

BRENDAH KATUGUME

0761251221/0741694346

[brendahkatugume2000@gmail.com](mailto:brendahkatugume2000@gmail.com)

Kyegegwa Uganda.

24/08/2024.

RE: RESPONSE TO REVIEWERS.

Dear Sir/madam,

I hereby write to present my gratitude to you for the valuable comments towards improving the research paper. We are sorry about the mistakes in our manuscript. According to the comments given by reviewer #4, we have attached the revised version of the manuscript and hope that these revisions and the improved text will be satisfactory.

| Journal requirements | My responses |
| --- | --- |
| 1. Thank you for submitting your revised manuscript. After careful consideration, we feel that it has merit but does not fully meet PLOS ONE’s publication criteria as it currently stands. Therefore, we invite you to submit a revised version of the manuscript that addresses the points raised during the review process. | Am glad that the journal recognizes our work and appreciates us for choosing it to publish it. As we were invited, we have attached a revised copy of the manuscript that addresses the points that were raised. |
| 2. Please submit your revised manuscript by Sep 09 2024 11:59PM. If you will need more time than this to complete your revisions, please reply to this message or contact the journal office at [plosone@plos.org](mailto:plosone@plos.org). When you're ready to submit your revision, log on to <https://www.editorialmanager.com/pone/> and select the 'Submissions Needing Revision' folder to locate your manuscript file. | Am glad that I was allowed all this long to respond back to the points that were raised for the betterment of our work, thank you so much. |
| 3. Please include the following items when submitting your revised manuscript:   - A rebuttal letter that responds to each point raised by the academic editor and reviewer(s). You should upload this letter as a separate file labelled 'Response to Reviewers'. - A marked-up copy of your manuscript that highlights changes made to the original version. You should upload this as a separate file labelled 'Revised Manuscript with Track Changes'. - An unmarked version of your revised paper without tracked changes. You should upload this as a separate file labeled 'Manuscript'. | A rebuttal letter has been attached as Word document labeled ‘Response to Reviewer’  A marked copy of the manuscript has been attached labelled ‘Revised Manuscript with Track Changes’  A new version of the manuscript has been attached to as a document labeled ‘Manuscript’. |
| 4.  If the authors have adequately addressed your comments raised in a previous round of review and you feel that this manuscript is now acceptable for publication, you may indicate that here to bypass the “Comments to the Author” section, enter your conflict of interest statement in the “Confidential to Editor” section, and submit your "Accept" recommendation.  Reviewer #3: All comments have been addressed  Reviewer #4: (No Response) | Thank you so much for the comment. |
| 5. Is the manuscript technically sound, and do the data support the conclusions?  The manuscript must describe a technically sound piece of scientific research with data that supports the conclusions. Experiments must have been conducted rigorously, with appropriate controls, replication, and sample sizes. The conclusions must be drawn appropriately based on the data presented.  Reviewer #3: Yes  Reviewer #4: No | Thank you so much for the comment. |
| 6.  Has the statistical analysis been performed appropriately and rigorously?  Reviewer #3: Yes  Reviewer #4: No | Am glad about your comments. |
| Have the authors made all data underlying the findings in their manuscript fully available?  The [PLOS Data policy](http://www.plosone.org/static/policies.action#sharing) requires authors to make all data underlying the findings described in their manuscript fully available without restriction, with rare exception (please refer to the Data Availability Statement in the manuscript PDF file). The data should be provided as part of the manuscript or its supporting information, or deposited to a public repository. For example, in addition to summary statistics, the data points behind means, medians and variance measures should be available. If there are restrictions on publicly sharing data—e.g. participant privacy or use of data from a third party—those must be specified.  Reviewer #3: Yes.  Reviewer #4: No. | Thank you so much for the response. |
| 5. Is the manuscript presented in an intelligible fashion and written in standard English?  PLOS ONE does not copyedit accepted manuscripts, so the language in submitted articles must be clear, correct, and unambiguous. Any typographical or grammatical errors should be corrected at revision, so please note any specific errors here.  Reviewer #3: Yes  Reviewer #4: No | Thank you so much for the response. |
| Review Comments to the Author  Please use the space provided to explain your answers to the questions above. You may also include additional comments for the author, including concerns about dual publication, research ethics, or publication ethics. (Please upload your review as an attachment if it exceeds 20,000 characters)  Reviewer #3: I have read through the responses to the comments and questions I raised. I am satisfied with the responses. | We deeply appreciate your response and the points which you raised before that made us improve our work. Thank you so much. |
| Reviewer #4: This study attempts to better understand the characteristics of neonatal sepsis at a single facility in Uganda. Specifically, they calculate the proportion of NICU admissions that are due to sepsis, and then use these data to do a variety of statistical tests. Overall, there is some interesting data here. I think the proportion of sepsis cases has implications for resources (human and material).  The authors suggest the sepsis prevalence of 41% is high, yet this primarily a function of non-sepsis related causes of admission and not the burden of sepsis per se. This might be 80 sepsis cases out of 10,000 babies born, which would make sepsis a very rare event, or 80 out of 300 births, which would make it a very common event. The authors do not have this data and thus cannot comment on sepsis prevalence. What these results do show, however, is that sepsis makes up nearly half of the NICU admissions, which is importance from a resource standpoint. this is what needs to be emphasized. b The referenced data from ref # 7 is itself referenced from another study. The authors should reference the study ($10 - $469 billion impact of NS) which actually did these analyses (which is referenced in ref #7)  What does LONS mean? This is on line 229.  There remain lots of grammatical errors (no spaces after periods, missing periods, incorrect capitalization, etc.) These needs to be fixed.  The formula for sample size calculation does not really make much sense, since the 498 number is arbitrary (based on the # of months of observation). If you had included charts from Jan - Dec, this number would differ. The main issue is the error around the estimated prevalence. b There was not a clear definition of sepsis, which is perhaps the most important piece in this study. How exactly was sepsis defined, what data was needed for this, and how much missingness of data was there?  in your tables, frequency should imply rate, not a pure number. Instead it should read n (%) and be in a single column  Table 4 is mixed up, with rows not aligning properly  what is the purpose of the multivariable analysis? Is it prediction or causation? These are very different and its not clear the purpose.  You need to define COR and AOR (crude vs adjusted I assume?)  The results describe the modelling process, but this is a method not a result  What was the mortality among the sepsis cases? Why was mortality not an outcome in the analysis of risk factors?  How many admissions were "rule out sepsis" vs established sepsis?  The issue with your final sample of 396 vs the intended 498 is not that you did not achieve your desired sample, but rather that the missing 100 subjects might significantly skew your results. this is an important potential bias. Missing information ALWAYs has the potential to affect the quality of the results, it has nothing to do with p-values per se. | We deeply appreciate you for commending our work.  This has been amended by editing the result reporting the prevalence as a proportion instead of a percentage. This further makes our results more relevant. As well the study only was considering the NICU admissions and comparing the results to all live births would make the study ambiguous.Thank you so much.  Thank you for the comment.  The mother reference of ref#7 was so long time ago which would make our work outdated, therefore the statement was withdrawn.  Thank you for the comment.  This has been amended by adding the full form of it to give it meaning.  This has been addressed, thank you for the comment  Thank you for the comment. The study considered 6 months which are also significant for a survey  Thank you for the comment. The definition of sepsis has been added.  Thank you for the comment. This has been addressed in all the tables with frequency.  This had been organized.  Multivariable analysis was used to find out the relationships between sepsis and the factors that were considered in the study.  Thank you for the comment. The definitions of COR and AOR have been added in the list of definitions  Thank you for the comment.  This was amended.  Thank you for the comment, however, depending on the objectives of the study, the outcome of sepsis was not part and so was one of the recommended points of research.  Thank you for the comment. This was explained in the manuscript. Considering the exclusion criteria, all records with missing information were to be left out of the study which quite affected the number of records used. This however was countered by response rate of above 50% which was 87.4% which gave us confidence to continue with the study since the response rate was above the average |

I will be grateful if my views, explanations and reservations are put under positive considerations.

Yours faithfully,

BRENDAH KATUGUME.

PRINCIPAL INVESTIGATOR.
