# Supplementary material for: Prevalence of neonatal sepsis and associated factors among neonates admitted in the neonatal intensive care unit at Lira Regional Referral Hospital, Northern Uganda
Source: PLoS One. 2025 Jan 13;20(1):e0315794. doi: 10.1371/journal.pone.0315794 (PMC11730428; doi:10.1371/journal.pone.0315794)
Supplement: Attachment — Submitted filename: RESPONSE TO REVIEWER.docx [file pone.0315794.s005.docx]

BRENDAH KATUGUME

0761251221/0741694346

[brendahkatugume2000@gmail.com](mailto:brendahkatugume2000@gmail.com)

Kyegegwa Uganda.

19/11/2024.

RE: RESPONSE TO REVIEWERS.

Dear Sir/Madam,

I hereby write to express my gratitude to you for the valuable comments towards improving the research paper. We are sorry about the mistakes in our manuscript. According to the comments given by reviewer #4, we have attached the revised version of the manuscript and hope that these revisions and the improved text will be satisfactory.

| Journal requirements | My responses |
| --- | --- |
| 1. Thank you for submitting your revised manuscript. After careful consideration, we feel that it has merit but does not fully meet PLOS ONE’s publication criteria as it currently stands. Therefore, we invite you to submit a revised version of the manuscript that addresses the points raised during the review process. | Am glad that the journal recognizes our work and appreciates us for choosing it to publish it. As we were invited, we have attached a revised copy of the manuscript that addresses the points that were raised. |
| 2. Please submit your revised manuscript by Nov 21 2024 11:59PM. If you will need more time than this to complete your revisions, please reply to this message or contact the journal office at [plosone@plos.org](mailto:plosone@plos.org). When you're ready to submit your revision, log on to <https://www.editorialmanager.com/pone/> and select the 'Submissions Needing Revision' folder to locate your manuscript file. | Am glad that I was allowed all this long to respond back to the points that were raised for the betterment of our work, thank you so much. |
| 3. Please include the following items when submitting your revised manuscript:   - A rebuttal letter that responds to each point raised by the academic editor and reviewer(s). You should upload this letter as a separate file labelled 'Response to Reviewers'. - A marked-up copy of your manuscript that highlights changes made to the original version. You should upload this as a separate file labelled 'Revised Manuscript with Track Changes'. - An unmarked version of your revised paper without tracked changes. You should upload this as a separate file labelled 'Manuscript'. | A rebuttal letter has been attached as Word document labeled ‘Response to Reviewer’  A marked copy of the manuscript has been attached labelled ‘Revised Manuscript with Track Changes’  A new version of the manuscript has been attached to as a document labeled ‘Manuscript’. |
| 4. If you would like to make changes to your financial disclosure, please include your updated statement in your cover letter. Guidelines for resubmitting your figure files are available below the review comments at the end of this letter. | Thank you so much for the comment. I don’t have any financial disclosure. |
| 5.  If the authors have adequately addressed your comments raised in a previous round of review and you feel that this manuscript is now acceptable for publication, you may indicate that here to bypass the “Comments to the Author” section, enter your conflict-of-interest statement in the “Confidential to Editor” section, and submit your "Accept" recommendation.  Reviewer #4: (No Response) | Thank you so much for the comment. |
| 6. Is the manuscript technically sound, and do the data support the conclusions? The manuscript must describe a technically sound piece of scientific research with data that supports the conclusions. Experiments must have been conducted rigorously, with appropriate controls, replication, and sample sizes. The conclusions must be drawn appropriately based on the data presented.  Reviewer #4: partly. | Thank you so much for the comment. We hope to make it fully technically sound. |
| 6.  Has the statistical analysis been performed appropriately and rigorously?  Reviewer #4: Yes | Am glad about your comment. |
| 7. Have the authors made all data underlying the findings in their manuscript fully available? The [PLOS Data policy](http://www.plosone.org/static/policies.action#sharing) requires authors to make all data underlying the findings described in their manuscript fully available without restriction, with rare exceptions (please refer to the Data Availability Statement in the manuscript PDF file). The data should be provided as part of the manuscript or its supporting information, or deposited to a public repository. For example, in addition to summary statistics, the data points behind means, medians, and variance measures should be available. If there are restrictions on publicly sharing data—e.g. participant privacy or use of data from a third party—those must be specified.  Reviewer #4: yes | Thank you so much for the response. |
| 5. Is the manuscript presented in an intelligible fashion and written in standard English?  PLOS ONE does not copyedit accepted manuscripts, so the language in submitted articles must be clear, correct, and unambiguous. Any typographical or grammatical errors should be corrected at revision, so please note any specific errors here.  Reviewer #4: Yes | Thank you so much for the response. |
| Review Comments to the Author  Please use the space provided to explain your answers to the questions above. You may also include additional comments for the author, including concerns about dual publication, research ethics, or publication ethics. (Please upload your review as an attachment if it exceeds 20,000 characters)  Reviewer #4: the sentence in the introduction about 2824 neonatal sepsis and 2469 EONS and 946 LONS is a little confusing. Is the “complete entity” meant to capture both EONS and LONS? If so, why do these numbers not add up? | We deeply appreciate your response and the points which you raised before that made us improve our work. Thank you so much.  Thank you so much for the comment. This statement was eliminated because we could not track it from the paper from which it had been picked. And because its removal could not affect our work. |
| The sample size continues to be an issue. The 498 is an arbitrary number. You could just as easily used 12 months of data and have a different number. 498 is not the “population of neonates admitted to the NICU at LRRH” but merely a slice of this population. The sample of 222 is yet a sub-slice of this slice. The solution is merely to state that 6-month time frame was used from which a random sub-sample was selected (describe how the “random” child was selected). You might need to justify why 6-m window is appropriate (given seasonality, etc). The actual sample enrolled was based on convenience, and this is fine. | Thank you for the comment. This was explained in the manuscript. Considering the exclusion criteria, all records with missing information were to be left out of the study which quite affected the number of records used. This however was countered by a response rate of above 50% which was 87.4% which gave us confidence to continue with the study since the response rate was above the average |
| The authors did not define sepsis in the methods. What was the criteria, when reviewing the chart, that caused them to assign “sepsis” vs not sepsis. There must have been a process. I can assume that some have” rule out sepsis” and some have established sepsis. You need to specify this. | Thank you for commenting. The definition of sepsis was based on the publication by the Uganda Pediatric Association and that was explained in the manuscript. This has been highlighted in the manuscript with tracked changes. I hope it answers the query. Thank you. |
| Results: A proportion is always between 0 and 1. The first paragraph states a "proportion of 80" | Thank you so much for the comment. This has been corrected, and a calculation of proportions has been made in the manuscript. |
| Discussion: The first paragraph outlines how different studies considered different methodologies to obtain the diagnosis of NS. Unfortunately, the methods do not outline the approach in this study. I suggest adding this in. There is a lot of content that reflects the results in the discussion, perhaps too much. However, the implications of these data are relevant in areas that remain unaddressed. Nearly half of NICU admissions are due to sepsis. How does this fact impact training, resources, etc? If the authors were to have an opportunity to discuss these results with the minister of health, what would they tell her about what the results MEAN (NOT what the results ARE)? It's the meaning of the results that ought to form the bulk of the discussion. | Thank you so much for the comment. However, this had been added already and it is highlighted in color red in the revised manuscript with tracked changes. Thank you.  A simple paragraph indicating the meaning of the results has been added in the discussion section. Thank you. |

I will be grateful if my views, explanations, and reservations are put under positive consideration.

Yours faithfully,

BRENDAH KATUGUME.

PRINCIPAL INVESTIGATOR.
