# Supplementary material for: Prevalence of neonatal sepsis and associated factors among neonates admitted in the neonatal intensive care unit at Lira Regional Referral Hospital, Northern Uganda
Source: PLoS One. 2025 Jan 13;20(1):e0315794. doi: 10.1371/journal.pone.0315794 (PMC11730428; doi:10.1371/journal.pone.0315794)
Supplement: Peer review history

# Peer review: Decision Letter 0

**DOI:** 10.1371/journal.pone.0315794.r001

**Authors:** Sanjoy Kumer Dey

14 Dec 2023

PONE-D-23-33964Prevalence of neonatal sepsis and associated factors among neonates admitted in the neonatal intensive care unit at Lira Regional Referral Hospital, northern Uganda.PLOS ONE

Dear Dr. KATUGUME,

Thank you for submitting your manuscript to PLOS ONE. After careful consideration, we feel that it has merit but does not fully meet PLOS ONE’s publication criteria as it currently stands. Therefore, we invite you to submit a revised version of the manuscript that addresses the points raised during the review process. Please submit your revised manuscript by Jan 28 2024 11:59PM. If you will need more time than this to complete your revisions, please reply to this message or contact the journal office at plosone@plos.org. When you're ready to submit your revision, log on to https://www.editorialmanager.com/pone/ and select the 'Submissions Needing Revision' folder to locate your manuscript file.

We look forward to receiving your revised manuscript.

Kind regards,

Sanjoy Kumer Dey, M.D

Academic Editor

PLOS ONE

Journal Requirements:

1. Please ensure that your manuscript meets PLOS ONE's style requirements, including those for file naming. The PLOS ONE style templates can be found at 

https://journals.plos.org/plosone/s/file?id=wjVg/PLOSOne_formatting_sample_main_body.pdf and 

https://journals.plos.org/plosone/s/file?id=ba62/PLOSOne_formatting_sample_title_authors_affiliations.pdf

2. Thank you for stating the following in the Acknowledgments Section of your manuscript: 

"I would like to acknowledge the research supervisor Ms. Namutebi Deborah Andrinar for her unfailing effort to the success of the study. My sincere appreciation to my beloved research lecturers at Lira university Mr. Udho Samson, Dr Amir Kabunga and Dr Rita Matte.  I am grateful to Dr. Nelson Okello and Dr. John Baptist Muzungu for the helping hand rendered in development of the data extraction tool. Am grateful to the department of midwifery, Faculty of Nursing and Midwifery and Lira University at large, LUREC, staff of NICU of LRRH and the whole administration of the hospital for enabling the study to be conducted. Furthermore, I am grateful to Mr. Eustes Kigongo for helping me understand and do data entry and analysis, friends Namawuba Vanessa Ruth and Ssuuna Thomasmoore for encouraging me and finally acknowledge my mother Ms. Kabatooro Rosemary for the unfailing financial support to the success of the study."

We note that you have provided funding information that is not currently declared in your Funding Statement. However, funding information should not appear in the Acknowledgments section or other areas of your manuscript. We will only publish funding information present in the Funding Statement section of the online submission form. 

Please remove any funding-related text from the manuscript and let us know how you would like to update your Funding Statement. Currently, your Funding Statement reads as follows: 

"The author(s) received no specific funding for this work."

Please include your amended statements within your cover letter; we will change the online submission form on your behalf.

3. In your Data Availability statement, you have not specified where the minimal data set underlying the results described in your manuscript can be found. PLOS defines a study's minimal data set as the underlying data used to reach the conclusions drawn in the manuscript and any additional data required to replicate the reported study findings in their entirety. All PLOS journals require that the minimal data set be made fully available. For more information about our data policy, please see http://journals.plos.org/plosone/s/data-availability.

Upon re-submitting your revised manuscript, please upload your study’s minimal underlying data set as either Supporting Information files or to a stable, public repository and include the relevant URLs, DOIs, or accession numbers within your revised cover letter. For a list of acceptable repositories, please see http://journals.plos.org/plosone/s/data-availability#loc-recommended-repositories. Any potentially identifying patient information must be fully anonymized.

Important: If there are ethical or legal restrictions to sharing your data publicly, please explain these restrictions in detail. Please see our guidelines for more information on what we consider unacceptable restrictions to publicly sharing data: http://journals.plos.org/plosone/s/data-availability#loc-unacceptable-data-access-restrictions. Note that it is not acceptable for the authors to be the sole named individuals responsible for ensuring data access.

We will update your Data Availability statement to reflect the information you provide in your cover letter.

4. We notice that your supplementary figures are uploaded with the file type 'Figure'. Please amend the file type to 'Supporting Information'. Please ensure that each Supporting Information file has a legend listed in the manuscript after the references list.

5. Please upload a copy of Figure 1, to which you refer in your text on page 10. If the figure is no longer to be included as part of the submission please remove all reference to it within the text.

[Note: HTML markup is below. Please do not edit.]

Reviewers' comments:

Reviewer's Responses to Questions

**Comments to the Author**

1. Is the manuscript technically sound, and do the data support the conclusions?

The manuscript must describe a technically sound piece of scientific research with data that supports the conclusions. Experiments must have been conducted rigorously, with appropriate controls, replication, and sample sizes. The conclusions must be drawn appropriately based on the data presented. 

Reviewer #1: Yes

Reviewer #2: No

**********

2. Has the statistical analysis been performed appropriately and rigorously? 

Reviewer #1: Yes

Reviewer #2: No

**********

3. Have the authors made all data underlying the findings in their manuscript fully available?

The PLOS Data policy requires authors to make all data underlying the findings described in their manuscript fully available without restriction, with rare exception (please refer to the Data Availability Statement in the manuscript PDF file). The data should be provided as part of the manuscript or its supporting information, or deposited to a public repository. For example, in addition to summary statistics, the data points behind means, medians and variance measures should be available. If there are restrictions on publicly sharing data—e.g. participant privacy or use of data from a third party—those must be specified.

Reviewer #1: Yes

Reviewer #2: No

**********

4. Is the manuscript presented in an intelligible fashion and written in standard English?

PLOS ONE does not copyedit accepted manuscripts, so the language in submitted articles must be clear, correct, and unambiguous. Any typographical or grammatical errors should be corrected at revision, so please note any specific errors here.

Reviewer #1: Yes

Reviewer #2: No

**********

5. Review Comments to the Author

Please use the space provided to explain your answers to the questions above. You may also include additional comments for the author, including concerns about dual publication, research ethics, or publication ethics. (Please upload your review as an attachment if it exceeds 20,000 characters)

Reviewer #1: I would like to thank the authors for investigating such an important topic that is relevant to clinical practice and the improvement of neonatal health.

Below are comments regarding the manuscript:

General concerns

The study is good; however, some major review is needed to improve it.

The work needs editing to improve the sentence construction and typing errors

The whole text needs sentence numbering, this will make review and comments easy

Abstract

Introduction:

Could there be statistics for sub-Saharan Africa other than relating your study to Global estimates? Global estimates fit the background section in the main text.

Your objective talks about the Lango sub-region; however, your study was carried out at Lira Regional Referral Hospital. Can the result represent the sub-region?

Methods

I think “A hospital-based cross-sectional study with a retrospective chart review was conducted….” Leaving out quantitative

The sentences are too long. This concerns the whole manuscript. Try to shorten them.

Also, you need not begin a sentence with “using”

Results:

Make the adjusted odds ratio (AOR) and confidence interval (CI) uniform across all the variables presented

Conclusion

The recommendation is broad. Are you saying all newborns should receive prophylaxis?

Background

This section is quite long. I would suggest you reduce the text.

Kindly number the sentences. This can be done from the Word document (layout).

Keep the sentences short, simple and clear.

The whole of the third paragraph is not cited. Also, the last 4 lines of this paragraph can be removed

The last 5 lines of the last paragraph are not for this section, take it to the method section.

Methods and materials

Study area

Any idea, how many babies are managed in the NICU annually

Population

Inclusion and exclusion criteria are missing

Ethics approval and consent to participate

You may need to edit the paragraph. Remove some details that are not very necessary. You may consult articles for guidance.

Data collection and quality control

This section has no information on the data collection procedure. It is mainly a description of the research instrument.

Please explain how the data were collected.

Also, you may need to define the variables considered and how they were measured. For instance, how was the gestation age determined? Also, how did you arrive at the diagnosis of neonatal sepsis?

Data processing and analysis

This paragraph also needs editing. Some sentences are not necessary e.g. data analysis was done at….

“Bivariate analysis was performed using…….” This sentence is not clear

Also, when you say “bivariate” what do you mean? I guess you mean bivariable

The backward elimination method was performed….. when was this done and why?

Confounding and interactions were assessed………. What was the result of your assessment?

How did you control for confounding?

Results

This section also needs editing and removing some paragraphs.

You may consider being brief and straight to the point.

In the results, readers want to see

a) how many charts (neonates) provided information for this study? Or the number of neonatal information/records reviewed. What were the proportions of your outcome (neonatal sepsis) and other conditions if available, and descriptions of the neonates and maternal (characteristics)? A piece of brief information is explained in a narrative while details are included in a table

b) factors associated with neonatal sepsis. Here you pick out variables that were significant while including their adjusted Odds ratio and confidence interval. No need to dedicate a whole paragraph to bivariable analysis. This can remain on the table.

You can construct a table with both crude and adjusted odds ratios, p values etc.

Change title from maternal factors to maternal characteristics

The title “bivariate…… of factors…., makes it factors associated with neonatal sepsis, indicated in (b) above

Tables: please put the title at the top of the table, and align figures with the variable name. For instance, Table 4: factors associated with neonatal sepsis among neonates admitted at NICU, LRRH. Edit all the tables.

In the text, you may not include P values. The readers can see from the table.

Check the variable names in Table 5

Kindly include variable names and their description in the method section so that one can make meaning of your findings.

Generally, improve on the presentation of your results. You may consult some articles for guidance.

Please, present the outcome of both bivariable and multivariable analysis in one table, if possible, with crude and adjusted OR and the P value of the adjusted OR.

Discussion

It's advisable not to discuss or explain other people’s studies while relating to your study.

You have not mentioned how the sepsis was diagnosed in the methods. Now you have included it in the discussion.

Your discussion mainly has studies from Ethiopia, do you mean studies conducted in Uganda are scarce?

Here you mention low birth weight. According to your data. Who are they?

It is common to find facts with no citation in this section. Kindly cite information that is not yours.

What were the strengths and limitations of this study?

A conclusion is absent. You need to include it.

Figure

You may need to use comparative colours to give a difference in the pie chart. (Sepsis and no sepsis). Make one lighter and another one darker.

The figure should have a label below

Remove the title inside the figure

Reviewer #2: Thanks you for reviewing your articles entitled Prevalence of neonatal sepsis and associated factors among neonates admitted in the neonatal intensive care unit at Lira Regional Referral Hospital, northern Uganda.

I have some comments and suggestions

Abstract part

* It says 80% have neonatal sepsis. At the same time it says 41%. So which one is correct?

* according to this study the APGAR score of 1-4 is protective.. how low APGAR score become protective raises the questions on the validity of the study. In this case it need resuscitation and that may be risk for the sepsis as many evidence suggested. Why your study is differ is not clear.

* Over all the result part need to rewrite.

Introduction

Need to use more updated data.

. Justification for conducted this particular was not acceptable

Methods and Materials

1. The research lack study population, inclusion and Exclusion Criteria.

2. Ethical consideration is also other issues. 2.1 it should be after data processing and analysis.

2.3 it should be short and to the point.

2.3. There is no any verb indicated you did not collect personal identifiers.

3 Sample size, method and techniques

3.1 Sample size calculation is not clear. I doubt its scientific.

3.2 Why you didn't used simple random sampling as your study was retrospective?

3.3 you said "198 respondents record " did you really have respondents?

3.4 which number was sellected from 1 to 2 in the first.

3.5 it say 198 and 194 so which one is the correct one?

4. Data collection process

4.1 Who were your data collectors

4.2 why you prefer UPA tool than any other standard tools.

4.3 how you keep quality of your research

4.4. How you measured your outcome variable

4.5 there are numerous problems in grammar and the chronological order to scientific papers. It look like the short note writing.

4.6 there is no dependent and independent variables. And how to measure outcomes variables was missed from the study.

4.7 ita said data collection tools taken from UPA and it also said. Quality was controlled by using other study tools. Please which one is correct?

4.8 do you think this is enough to control your the quality of your research.

5. Data analysis methods

5.1 Need to rewrite. Because it says *(p-value <0.20 used to get candidates for multivariate analysis. Again it say backwards stepwise methods was used" I confused with such analysis?.. I think it needs to rewrite or to stick either of the two.

5.2. How you control confound and interaction should be reported.

5.2 overall it need to rewrite again.

6.1 Repetition of idea in the first 7 line of the result should be summerized.

6.2 majority of neonates were low birth weight could you give us justification ?

6.3 many important variables of the maternal missed

6.4 in the table APGAR 8_10 ---no observation reported, why?

6.7.if you observe your cross tab indicates most of neonates with normal birth, with resuscitation , low APGAR score have high sepsis prevalence. Why???

6.8 check APGAR score it say April......what you mean that??

6.8. Many variables missed

6.8. Merge bivariate and multivariate analysis.

6.9 I have doubt that the method of data analy was no well implemented

7. Discussion

Not well organised

8. The research lacks many important components: strengths, limitations, conclusion and recommendations .

**********

6. PLOS authors have the option to publish the peer review history of their article (what does this mean?). If published, this will include your full peer review and any attached files.

If you choose “no”, your identity will remain anonymous but your review may still be made public.

**Do you want your identity to be public for this peer review?** For information about this choice, including consent withdrawal, please see our Privacy Policy.

Reviewer #1: No

Reviewer #2: No

**********

[NOTE: If reviewer comments were submitted as an attachment file, they will be attached to this email and accessible via the submission site. Please log into your account, locate the manuscript record, and check for the action link "View Attachments". If this link does not appear, there are no attachment files.]

While revising your submission, please upload your figure files to the Preflight Analysis and Conversion Engine (PACE) digital diagnostic tool, https://pacev2.apexcovantage.com/. PACE helps ensure that figures meet PLOS requirements. To use PACE, you must first register as a user. Registration is free. Then, login and navigate to the UPLOAD tab, where you will find detailed instructions on how to use the tool. If you encounter any issues or have any questions when using PACE, please email PLOS at figures@plos.org. Please note that Supporting Information files do not need this step.

# Author comment: Author response to Decision Letter 0

**DOI:** 10.1371/journal.pone.0315794.r002

1 Feb 2024

good afternoon, sir.

I am glad to have received your feedback about the manuscript and am glad to inform you that I have personally responded to all comments and attached a revised copy of the manuscript.

kindly 

katugume Brendah

principal investigator

Attachment Submitted filename: RESPONSE TO REVIEWER.docx

# Peer review: Decision Letter 1

**DOI:** 10.1371/journal.pone.0315794.r003

**Authors:** Sanjoy Kumer Dey

7 Mar 2024

PONE-D-23-33964R1Prevalence of neonatal sepsis and associated factors among neonates admitted in the neonatal intensive care unit at Lira Regional Referral Hospital, northern Uganda.PLOS ONE

Dear Dr. KATUGUME,

Thank you for submitting your manuscript to PLOS ONE. After careful consideration, we feel that it has merit but does not fully meet PLOS ONE’s publication criteria as it currently stands. Therefore, we invite you to submit a revised version of the manuscript that addresses the points raised during the review process.

Please submit your revised manuscript by Apr 21 2024 11:59PM. If you will need more time than this to complete your revisions, please reply to this message or contact the journal office at plosone@plos.org. When you're ready to submit your revision, log on to https://www.editorialmanager.com/pone/ and select the 'Submissions Needing Revision' folder to locate your manuscript file.

Please include the following items when submitting your revised manuscript:

- A rebuttal letter that responds to each point raised by the academic editor and reviewer(s). You should upload this letter as a separate file labeled 'Response to Reviewers'.
- A marked-up copy of your manuscript that highlights changes made to the original version. You should upload this as a separate file labeled 'Revised Manuscript with Track Changes'.
- An unmarked version of your revised paper without tracked changes. You should upload this as a separate file labeled 'Manuscript'.

If you would like to make changes to your financial disclosure, please include your updated statement in your cover letter. Guidelines for resubmitting your figure files are available below the reviewer comments at the end of this letter.

If applicable, we recommend that you deposit your laboratory protocols in protocols.io to enhance the reproducibility of your results. Protocols.io assigns your protocol its own identifier (DOI) so that it can be cited independently in the future. For instructions see: https://journals.plos.org/plosone/s/submission-guidelines#loc-laboratory-protocols. Additionally, PLOS ONE offers an option for publishing peer-reviewed Lab Protocol articles, which describe protocols hosted on protocols.io. Read more information on sharing protocols at https://plos.org/protocols?utm_medium=editorial-email&utm_source=authorletters&utm_campaign=protocols.

We look forward to receiving your revised manuscript.

Kind regards,

Sanjoy Kumer Dey, M.D

Academic Editor

PLOS ONE

Journal Requirements:

Please review your reference list to ensure that it is complete and correct. If you have cited papers that have been retracted, please include the rationale for doing so in the manuscript text, or remove these references and replace them with relevant current references. Any changes to the reference list should be mentioned in the rebuttal letter that accompanies your revised manuscript. If you need to cite a retracted article, indicate the article’s retracted status in the References list and also include a citation and full reference for the retraction notice.

[Note: HTML markup is below. Please do not edit.]

Reviewers' comments:

Reviewer's Responses to Questions

**Comments to the Author**

1. If the authors have adequately addressed your comments raised in a previous round of review and you feel that this manuscript is now acceptable for publication, you may indicate that here to bypass the “Comments to the Author” section, enter your conflict of interest statement in the “Confidential to Editor” section, and submit your "Accept" recommendation.

Reviewer #3: All comments have been addressed

**********

2. Is the manuscript technically sound, and do the data support the conclusions?

The manuscript must describe a technically sound piece of scientific research with data that supports the conclusions. Experiments must have been conducted rigorously, with appropriate controls, replication, and sample sizes. The conclusions must be drawn appropriately based on the data presented. 

Reviewer #3: Yes

**********

3. Has the statistical analysis been performed appropriately and rigorously? 

Reviewer #3: Yes

**********

4. Have the authors made all data underlying the findings in their manuscript fully available?

The PLOS Data policy requires authors to make all data underlying the findings described in their manuscript fully available without restriction, with rare exception (please refer to the Data Availability Statement in the manuscript PDF file). The data should be provided as part of the manuscript or its supporting information, or deposited to a public repository. For example, in addition to summary statistics, the data points behind means, medians and variance measures should be available. If there are restrictions on publicly sharing data—e.g. participant privacy or use of data from a third party—those must be specified.

Reviewer #3: Yes

**********

5. Is the manuscript presented in an intelligible fashion and written in standard English?

PLOS ONE does not copyedit accepted manuscripts, so the language in submitted articles must be clear, correct, and unambiguous. Any typographical or grammatical errors should be corrected at revision, so please note any specific errors here.

Reviewer #3: Yes

**********

6. Review Comments to the Author

Please use the space provided to explain your answers to the questions above. You may also include additional comments for the author, including concerns about dual publication, research ethics, or publication ethics. (Please upload your review as an attachment if it exceeds 20,000 characters)

Reviewer #3: Thank you for inviting me to review this manuscript titled “Prevalence of neonatal sepsis and associated factors among neonates admitted in the neonatal intensive care unit at Lira Regional Referral Hospital, northern Uganda”

Neonatal sepsis is a leading cause of neonatal morbidity and mortality in the low resource settings. The concept was well though and the importance of the area of study cannot be underestimated. The prevalence was alarmingly high!

There’s generally need to correct the numerous typographical and grammatical errors much as these errors do not adversely affect the results. Also be consistent in the line spacing and font type and size!

Abstract: You mention that the study was done in the Lango sub-region. Is Lira hospital, which was the epicentre of the study, representative of the Lango sub-region? If not, please state exactly where the study was done i.e Lira regional referral hospital!

Introduction: The introduction was well written and contains relevant information to the study. There is however a mix in the referencing/citation style. Please stick to one referencing style and be consistent

Methods and materials: There’s need to mention or write a sentence or two on the capacity of Lira Hospital in regard to neonatal care services and the NICU. This will give the reader an insight into the justification for the study at that site. The rest of the methods section is easy to follow through!

Results: The results are informative. Table 5 on factors associated with neonatal sepsis needs to be complete by including all the p-values and ORs for the variables listed!

Discussion: There’s some attempt to explain the comparisons between the study and studies cited. The recommendations need to be directed to entities that the author (s) believe will be able to drive the recommendation.

**********

7. PLOS authors have the option to publish the peer review history of their article (what does this mean?). If published, this will include your full peer review and any attached files.

If you choose “no”, your identity will remain anonymous but your review may still be made public.

**Do you want your identity to be public for this peer review?** For information about this choice, including consent withdrawal, please see our Privacy Policy.

Reviewer #3: No

**********

[NOTE: If reviewer comments were submitted as an attachment file, they will be attached to this email and accessible via the submission site. Please log into your account, locate the manuscript record, and check for the action link "View Attachments". If this link does not appear, there are no attachment files.]

While revising your submission, please upload your figure files to the Preflight Analysis and Conversion Engine (PACE) digital diagnostic tool, https://pacev2.apexcovantage.com/. PACE helps ensure that figures meet PLOS requirements. To use PACE, you must first register as a user. Registration is free. Then, login and navigate to the UPLOAD tab, where you will find detailed instructions on how to use the tool. If you encounter any issues or have any questions when using PACE, please email PLOS at figures@plos.org. Please note that Supporting Information files do not need this step.

# Author comment: Author response to Decision Letter 1

**DOI:** 10.1371/journal.pone.0315794.r004

19 Apr 2024

BRENDAH KATUGUME

+256 761 251 221/ 741 694 346

brendahkatugume2000@gmail.com

Kyegegwa Uganda.

19/04/2024.

TO THE ACADEMIC EDITOR, 

PLOS ONE JOURNAL

Dear Dr.

RE: RESPONSE TO REVIEWER COMMENTS.

I hereby write to appreciate you doctor and the journal at large for considering our work for review, the reviewers as well for giving us very important comments aimed at improving the quality of our work.

This letter is to inform you that I have duly received and appreciated the manuscript evaluation report of the study entitled Prevalence of neonatal sepsis and associated factors among neonates admitted to NICU of LRRH given on 7th of March 2024 at 05:13 Am. Although I agree with some of the evaluations, I shall respectfully make some reservations.

S/NO REVIEWER’S COMMENT AUTHOR’S RESPONSE PAGE NUMBER

1. Neonatal sepsis is a leading cause of neonatal morbidity and mortality in the low resource settings. The concept was well though and the importance of the area of study cannot be underestimated. The prevalence was alarmingly high! Thank you for commending our work.

2. There’s generally a need to correct the numerous typographical and grammatical errors much as these errors do not adversely affect the results Thank you for considering our work, the grammar of the document has been corrected as indicated in the revised manuscript with tracked changes. The entire document.

3. Also, be consistent in the line spacing and font type and size! Thank you so much, this too has been addressed. The whole document.

4. Abstract: You mention that the study was done in the Lango sub-region. Is Lira hospital, which was the epicentre of the study, representative of the Lango sub-region? If not, please state exactly where the study was done i.e Lira regional referral hospital! Am glad about the comment. the study area was edited and instead of lango subregion it was narrowed back to Lira Regional Referral Hospital with an explanation of the study being conducted in LRRH only 1,2.

5. Introduction: The introduction was well written and contains relevant information to the study. There is however a mix in the referencing/citation style. Please stick to one referencing style and be consistent Thank you so much for pointing this out. The point of mixed citations was because citation was done with different referencing managers but that was edited and redone using the sme referencing manager through out the whole manuscript. 3,4.

6. Methods and materials: There’s need to mention or write a sentence or two on the capacity of Lira Hospital in regard to neonatal care services and the NICU. This will give the reader an insight into the justification for the study at that site. Am glad that i have been reminded about this, it too has been addressed. The capacity of the NICU was also included and that considered 2 years, 2021 and 2022. 5

7. The rest of the methods section is easy to follow through! Thank you for commending our work 

8. Results: The results are informative. We are grateful about the comment. 

9. Table 5 on factors associated with neonatal sepsis needs to be complete by including all the p-values and ORs for the variables listed! Thank you for the comment. For this comment, no editing has been done to the table but the explanation has been provided. Part of the odds ratios of table 5 are missing because those particular variables were not considered for multivariable analysis since they had a p-value that was bigger than 0.2 which was significant for that level in data analysis. 14

10. Discussion: There’s some attempt to explain the comparisons between the study and studies cited Thank you for commending our work. 

11. The recommendations need to be directed to entities that the author (s) believe will be able to drive the recommendation. Thank you the comments. The point of having the recommendations being directed has been done too as per the tracked changes. 17

I will be grateful if my views, explanations and reservations are put under positive considerations.

Yours faithfully,

BRENDAH KATUGUME.

PRINCIPAL INVESTIGATOR.

Attachment Submitted filename: Response to reviewers..docx

# Peer review: Decision Letter 2

**DOI:** 10.1371/journal.pone.0315794.r005

**Authors:** Sanjoy Kumer Dey

26 Jul 2024

PONE-D-23-33964R2Prevalence of neonatal sepsis and associated factors among neonates admitted in the neonatal intensive care unit at Lira Regional Referral Hospital, northern Uganda.PLOS ONE

Dear Dr. KATUGUME, Thank you for submitting your revised manuscript. After careful consideration, we feel that it has merit but does not fully meet PLOS ONE’s publication criteria as it currently stands. Therefore, we invite you to submit a revised version of the manuscript that addresses the points raised during the review process.

Please submit your revised manuscript by Sep 09 2024 11:59PM. If you will need more time than this to complete your revisions, please reply to this message or contact the journal office at plosone@plos.org. When you're ready to submit your revision, log on to https://www.editorialmanager.com/pone/ and select the 'Submissions Needing Revision' folder to locate your manuscript file.

Please include the following items when submitting your revised manuscript:

- A rebuttal letter that responds to each point raised by the academic editor and reviewer(s). You should upload this letter as a separate file labeled 'Response to Reviewers'.
- A marked-up copy of your manuscript that highlights changes made to the original version. You should upload this as a separate file labeled 'Revised Manuscript with Track Changes'.
- An unmarked version of your revised paper without tracked changes. You should upload this as a separate file labeled 'Manuscript'.

We look forward to receiving your revised manuscript.

Kind regards,

Sanjoy Kumer Dey, M.D

Academic Editor

PLOS ONE

[Note: HTML markup is below. Please do not edit.]

Reviewers' comments:

Reviewer's Responses to Questions

**Comments to the Author**

1. If the authors have adequately addressed your comments raised in a previous round of review and you feel that this manuscript is now acceptable for publication, you may indicate that here to bypass the “Comments to the Author” section, enter your conflict of interest statement in the “Confidential to Editor” section, and submit your "Accept" recommendation.

Reviewer #3: All comments have been addressed

Reviewer #4: (No Response)

**********

2. Is the manuscript technically sound, and do the data support the conclusions?

The manuscript must describe a technically sound piece of scientific research with data that supports the conclusions. Experiments must have been conducted rigorously, with appropriate controls, replication, and sample sizes. The conclusions must be drawn appropriately based on the data presented. 

Reviewer #3: Yes

Reviewer #4: No

**********

3. Has the statistical analysis been performed appropriately and rigorously? 

Reviewer #3: Yes

Reviewer #4: No

**********

4. Have the authors made all data underlying the findings in their manuscript fully available?

The PLOS Data policy requires authors to make all data underlying the findings described in their manuscript fully available without restriction, with rare exception (please refer to the Data Availability Statement in the manuscript PDF file). The data should be provided as part of the manuscript or its supporting information, or deposited to a public repository. For example, in addition to summary statistics, the data points behind means, medians and variance measures should be available. If there are restrictions on publicly sharing data—e.g. participant privacy or use of data from a third party—those must be specified.

Reviewer #3: Yes

Reviewer #4: No

**********

5. Is the manuscript presented in an intelligible fashion and written in standard English?

PLOS ONE does not copyedit accepted manuscripts, so the language in submitted articles must be clear, correct, and unambiguous. Any typographical or grammatical errors should be corrected at revision, so please note any specific errors here.

Reviewer #3: Yes

Reviewer #4: No

**********

6. Review Comments to the Author

Please use the space provided to explain your answers to the questions above. You may also include additional comments for the author, including concerns about dual publication, research ethics, or publication ethics. (Please upload your review as an attachment if it exceeds 20,000 characters)

Reviewer #3: I have read through the responses to the comments and questions I raised. I am satisfied with the responses.

Reviewer #4: This study attempts to better understand the characteristics of neonatal sepsis at a single facility in Uganda. Specifically they calculate the proportion of NICU admissions that are due to sepsis, and then use these data to do a variety of statistical tests. Overall, there is some interesting data here. I think the proportion of sepsis cases has implications for resources (human and material).

The authors suggest the sepsis prevalence of 41% is high, yet this primarily a function of non-sepsis related causes of admission and not the burden of sepsis per se. This might be 80 sepsis cases out of 10,000 babies born, which would make sepsis a very rare event, or 80 out of 300 births, which would make it a very common event. The authors do not have this data and thus cannot comment on sepsis prevalence. What these results do show, however, is that sepsis makes up nearly half of the NICU admissions, which is importance from a resource standpoint. this is what needs to be emphasized.

The referenced data from ref # 7 is itself referenced from another study. The authors should reference the study ($10 - $469 billion impact of NS) which actually did these analyses (which is referenced in ref #7)

What does LONS mean? This is on line 229.

There remain lots of grammatical errors (no spaces after periods, missing periods, incorrect capitalization, etc.) These needs to be fixed.

The formula for sample size calculation does not really make much sense, since the 498 number is arbitrary (based on the # of months of observation). If you had included charts from Jan - Dec, this number would differ. The main issue is the error around the estimated prevalence.

There was not a clear definition of sepsis, which is perhaps the most important piece in this study. How exactly was sepsis defined, what data was needed for this, and how much missingness of data was there?

in your tables, frequency should imply rate, not a pure number. Instead it should read n(%) and be in a single column

Table 4 is mixed up, with rows not aligning properly

what is the purpose of the multivariable analysis? Is it prediction or causation? These are very different and its not clear the purpose.

You need to define COR and AOR (crude vs adjusted I assume?)

The results describe the modelling process, but this is a method not a result

What was the mortality among the sepsis cases? Why was mortality not an outcome in the analysis of risk factors?

How many admissions were "rule out sepsis" vs established sepsis?

The issue with your final sample of 396 vs the intended 498 is not that you did not achieve your desired sample, but rather that the missing 100 subjects might significantly skew your results. this is an important potential bias. Missing information ALWAYs has the potential to affect the quality of the results, it has nothing to do with p-values per se.

**********

7. PLOS authors have the option to publish the peer review history of their article (what does this mean?). If published, this will include your full peer review and any attached files.

If you choose “no”, your identity will remain anonymous but your review may still be made public.

**Do you want your identity to be public for this peer review?** For information about this choice, including consent withdrawal, please see our Privacy Policy.

Reviewer #3: **Yes: **Joseph Ngonzi

Reviewer #4: No

**********

[NOTE: If reviewer comments were submitted as an attachment file, they will be attached to this email and accessible via the submission site. Please log into your account, locate the manuscript record, and check for the action link "View Attachments". If this link does not appear, there are no attachment files.]

While revising your submission, please upload your figure files to the Preflight Analysis and Conversion Engine (PACE) digital diagnostic tool, https://pacev2.apexcovantage.com/. PACE helps ensure that figures meet PLOS requirements. To use PACE, you must first register as a user. Registration is free. Then, login and navigate to the UPLOAD tab, where you will find detailed instructions on how to use the tool. If you encounter any issues or have any questions when using PACE, please email PLOS at figures@plos.org. Please note that Supporting Information files do not need this step.

# Author comment: Author response to Decision Letter 2

**DOI:** 10.1371/journal.pone.0315794.r006

8 Sep 2024

Thank you for the time you have incurred to make sure this paper becomes the best you have made it, we deeply appreciate and request you not to get tired of doing so. Thank you

Attachment Submitted filename: RESPONSE TO REVIEWER new.docx

# Peer review: Decision Letter 3

**DOI:** 10.1371/journal.pone.0315794.r007

**Authors:** Sanjoy Kumer Dey

7 Oct 2024

PONE-D-23-33964R3Prevalence of neonatal sepsis and associated factors among neonates admitted in the neonatal intensive care unit at Lira Regional Referral Hospital, northern Uganda.PLOS ONE

Dear Dr. KATUGUME,

Thank you for submitting your manuscript to PLOS ONE. After careful consideration, we feel that it has merit but does not fully meet PLOS ONE’s publication criteria as it currently stands. Therefore, we invite you to submit a revised version of the manuscript that addresses the points raised during the review process.

Please submit your revised manuscript by Nov 21 2024 11:59PM. If you will need more time than this to complete your revisions, please reply to this message or contact the journal office at plosone@plos.org. When you're ready to submit your revision, log on to https://www.editorialmanager.com/pone/ and select the 'Submissions Needing Revision' folder to locate your manuscript file.

Please include the following items when submitting your revised manuscript:

- A rebuttal letter that responds to each point raised by the academic editor and reviewer(s). You should upload this letter as a separate file labeled 'Response to Reviewers'.
- A marked-up copy of your manuscript that highlights changes made to the original version. You should upload this as a separate file labeled 'Revised Manuscript with Track Changes'.
- An unmarked version of your revised paper without tracked changes. You should upload this as a separate file labeled 'Manuscript'.

If you would like to make changes to your financial disclosure, please include your updated statement in your cover letter. Guidelines for resubmitting your figure files are available below the reviewer comments at the end of this letter.

We look forward to receiving your revised manuscript.

Kind regards,

Sanjoy Kumer Dey, M.D

Academic Editor

PLOS ONE

Journal Requirements:

Please review your reference list to ensure that it is complete and correct. If you have cited papers that have been retracted, please include the rationale for doing so in the manuscript text, or remove these references and replace them with relevant current references. Any changes to the reference list should be mentioned in the rebuttal letter that accompanies your revised manuscript. If you need to cite a retracted article, indicate the article’s retracted status in the References list and also include a citation and full reference for the retraction notice.

[Note: HTML markup is below. Please do not edit.]

Reviewers' comments:

Reviewer's Responses to Questions

**Comments to the Author**

1. If the authors have adequately addressed your comments raised in a previous round of review and you feel that this manuscript is now acceptable for publication, you may indicate that here to bypass the “Comments to the Author” section, enter your conflict of interest statement in the “Confidential to Editor” section, and submit your "Accept" recommendation.

Reviewer #4: (No Response)

**********

2. Is the manuscript technically sound, and do the data support the conclusions?

The manuscript must describe a technically sound piece of scientific research with data that supports the conclusions. Experiments must have been conducted rigorously, with appropriate controls, replication, and sample sizes. The conclusions must be drawn appropriately based on the data presented. 

Reviewer #4: Partly

**********

3. Has the statistical analysis been performed appropriately and rigorously? 

Reviewer #4: Yes

**********

4. Have the authors made all data underlying the findings in their manuscript fully available?

The PLOS Data policy requires authors to make all data underlying the findings described in their manuscript fully available without restriction, with rare exception (please refer to the Data Availability Statement in the manuscript PDF file). The data should be provided as part of the manuscript or its supporting information, or deposited to a public repository. For example, in addition to summary statistics, the data points behind means, medians and variance measures should be available. If there are restrictions on publicly sharing data—e.g. participant privacy or use of data from a third party—those must be specified.

Reviewer #4: Yes

**********

5. Is the manuscript presented in an intelligible fashion and written in standard English?

PLOS ONE does not copyedit accepted manuscripts, so the language in submitted articles must be clear, correct, and unambiguous. Any typographical or grammatical errors should be corrected at revision, so please note any specific errors here.

Reviewer #4: Yes

**********

6. Review Comments to the Author

Please use the space provided to explain your answers to the questions above. You may also include additional comments for the author, including concerns about dual publication, research ethics, or publication ethics. (Please upload your review as an attachment if it exceeds 20,000 characters)

Reviewer #4: The sentence in the introduction about 2824 neonatal sepsis and 2469 EONS and 946 LONS is a little confusing. Is the "complete entity" meant to capture both EONS and LONS? If so, why do these numbers not add up?

The sample size continues to be an issue. The 498 is an arbitrary number. you could just as easily used 12 months of data and had a different number. 498 is not the "population of neonates admitted to the NICU at LRRH", but merely a slice of this population. The sample of 222 is yet a sub-slice of this slice. The solution is merely to state that a 6-month time frame was used from which a random sub-sample was selected (describe how the "random" child was selected). You might need to justify why a 6-m window is appropriate (given seasonality, etc.). The actual sample enrolled was based on convenience, and this is fine.

The authors did not define sepsis in the methods. What was the criteria, when reviewing the chart, that caused them to assign "sepsis" vs not sepsis. There must have been a process. I can assume that some have "rule out sepsis" and some have established sepsis. You need to specify this.

Results:

A proportion is always between 0 and 1. The first paragraph states a "proportion of 80"

Discussion:

The first paragraph outlines how different studies considered different methodologies to obtain the diagnosis of NS. Unfortunately the methods do not outline the approach in this study. I suggest adding this in.

There is a lot of content that reflects the results in the discussion, perhaps too much. However, the implications of these data are relevant in areas that remain unaddressed. Nearly half of NICU admissions are due to sepsis. How does this fact impact training, resources, etc? If the authors were to have an opportunity to discuss these results with the minster of health, what would they tell her about what the result MEAN (NOT what the results ARE)? Its the meaning of the results that ought to form the bulk of the discussion.

**********

7. PLOS authors have the option to publish the peer review history of their article (what does this mean?). If published, this will include your full peer review and any attached files.

If you choose “no”, your identity will remain anonymous but your review may still be made public.

**Do you want your identity to be public for this peer review?** For information about this choice, including consent withdrawal, please see our Privacy Policy.

Reviewer #4: No

**********

[NOTE: If reviewer comments were submitted as an attachment file, they will be attached to this email and accessible via the submission site. Please log into your account, locate the manuscript record, and check for the action link "View Attachments". If this link does not appear, there are no attachment files.]

While revising your submission, please upload your figure files to the Preflight Analysis and Conversion Engine (PACE) digital diagnostic tool, https://pacev2.apexcovantage.com/. PACE helps ensure that figures meet PLOS requirements. To use PACE, you must first register as a user. Registration is free. Then, login and navigate to the UPLOAD tab, where you will find detailed instructions on how to use the tool. If you encounter any issues or have any questions when using PACE, please email PLOS at figures@plos.org. Please note that Supporting Information files do not need this step.

# Author comment: Author response to Decision Letter 3

**DOI:** 10.1371/journal.pone.0315794.r008

19 Nov 2024

I am glad to present my gratitude to you all my dear reviewers, and I hope I have covered all the requirements. I look forward to seeing my paper published. thank you

Attachment Submitted filename: RESPONSE TO REVIEWER.docx

# Editor report: Decision Letter 4

**DOI:** 10.1371/journal.pone.0315794.r009

**Authors:** Sanjoy Kumer Dey

3 Dec 2024

Prevalence of neonatal sepsis and associated factors among neonates admitted in the neonatal intensive care unit at Lira Regional Referral Hospital, northern Uganda.

PONE-D-23-33964R4

Dear Dr. BRENDAH KATUGUME

We’re pleased to inform you that your manuscript has been judged scientifically suitable for publication and will be formally accepted for publication once it meets all outstanding technical requirements.

Within one week, you’ll receive an e-mail detailing the required amendments. When these have been addressed, you’ll receive a formal acceptance letter and your manuscript will be scheduled for publication.

An invoice will be generated when your article is formally accepted. Please note, if your institution has a publishing partnership with PLOS and your article meets the relevant criteria, all or part of your publication costs will be covered. Please make sure your user information is up-to-date by logging into Editorial Manager at Editorial Manager® and clicking the ‘Update My Information' link at the top of the page. If you have any questions relating to publication charges, please contact our Author Billing department directly at authorbilling@plos.org.

If your institution or institutions have a press office, please notify them about your upcoming paper to help maximize its impact. If they’ll be preparing press materials, please inform our press team as soon as possible -- no later than 48 hours after receiving the formal acceptance. Your manuscript will remain under strict press embargo until 2 pm Eastern Time on the date of publication. For more information, please contact onepress@plos.org.

Kind regards,

Sanjoy Kumer Dey, M.D

Academic Editor

PLOS ONE

Additional Editor Comments (optional):

Reviewers' comments:

# Editor report: Acceptance letter

**DOI:** 10.1371/journal.pone.0315794.r010

**Authors:** Sanjoy Kumer Dey

27 Dec 2024

PONE-D-23-33964R4 

PLOS ONE

Dear Dr. KATUGUME, 

I'm pleased to inform you that your manuscript has been deemed suitable for publication in PLOS ONE. Congratulations! Your manuscript is now being handed over to our production team.

At this stage, our production department will prepare your paper for publication. This includes ensuring the following:

* All references, tables, and figures are properly cited

* All relevant supporting information is included in the manuscript submission,

* There are no issues that prevent the paper from being properly typeset

If revisions are needed, the production department will contact you directly to resolve them. If no revisions are needed, you will receive an email when the publication date has been set. At this time, we do not offer pre-publication proofs to authors during production of the accepted work. Please keep in mind that we are working through a large volume of accepted articles, so please give us a few weeks to review your paper and let you know the next and final steps. 

Lastly, if your institution or institutions have a press office, please let them know about your upcoming paper now to help maximize its impact. If they'll be preparing press materials, please inform our press team within the next 48 hours. Your manuscript will remain under strict press embargo until 2 pm Eastern Time on the date of publication. For more information, please contact onepress@plos.org.

If we can help with anything else, please email us at customercare@plos.org.

Thank you for submitting your work to PLOS ONE and supporting open access. 

Kind regards, 

PLOS ONE Editorial Office Staff

on behalf of

Dr. Sanjoy Kumer Dey 

Academic Editor

PLOS ONE
